# Stimulation of Adult Muscle Stem Cells with BMPs Results in Direct Activation of Notch Pathway Genes

**DOI:** 10.3390/cells15161490

**Published:** 2026-08-19

**Authors:** Birthe Katrin Alexandra Lange, Ioanna Polydorou, Viktoriia Huryn, Angelina M. Georgieva, Shanshan You, Thomas Müller, Susanne Morales-Gonzalez, Bettina Brandt, Carmen Birchmeier, Helge Amthor, Markus Schuelke

**Affiliations:** 1 Department of Neuropediatrics, Charité—University Medical Center Berlin, Corporate Member of Freie Universität Berlin and Humboldt-Universität zu Berlin, Augustenburger Platz 1, 13353 Berlin, Germany; birthe.lange@charite.de (B.K.A.L.); ioanna.polydorou@protonmail.com (I.P.); susanne.morales-gonzalez@charite.de (S.M.-G.); 2 Department of Biology, Humboldt-Universität zu Berlin, Unter den Linden 6, 10099 Berlin, Germany; 3 NeuroCure Cluster of Excellence, Charité—University Medical Center Berlin, Corporate Member of Freie Universität Berlin and Humboldt-Universität zu Berlin, Charitéplatz 1, 10117 Berlin, Germany; angelina-momchilova.georgieva@charite.de (A.M.G.); shanshan.you@charite.de (S.Y.); thomas.mueller2@charite.de (T.M.); carmen.birchmeier@charite.de (C.B.); 4 U1179 Université de Versailles Saint-Quentin-en-Yvelines (UVSQ)—Institut National de la Santé et de la Recherche Médicale (INSERM), 78180 Montigny-le-Bretonneux, France; helge.amthor@aphp.fr; 5 Berlin Institute for Medical Systems Biology (BIMSB), Max-Delbrück-Center for Molecular Medicine in the Helmholtz Association (MDC), Hannoversche Str. 28, 10115 Berlin, Germany; viktoriia.huryn@mdc-berlin.de; 6 Department of Computer Science, Humboldt-Universität zu Berlin, Unter den Linden 6, 10099 Berlin, Germany; 7 Integrative Biomedicine, Max-Delbrück-Center for Molecular Medicine in the Helmholtz Association (MDC), 13125 Berlin, Germany; bettina.brandt@mdc-berlin.de; 8 Department of Biology, Chemistry and Pharmacy, Freie Universität Berlin, 14195 Berlin, Germany

**Keywords:** bone morphogenetic protein, Notch pathway, adult muscle stem cell, mouse, RNA-sequencing, transcription factor binding site, pSMAD1/5/9, SMAD4, CUT&Tag

## Abstract

**Highlights:**

**What are the main findings?**

- BMP6 stimulation upregulates classical Notch pathway genes.
- CUT&Tag after BMP6 stimulation identifies pSMAD1/5/9 and SMAD4 binding sites in promoters and regulatory regions of Notch and BMP pathway genes.

**What are the implications of the main findings?**

- A set of well-characterized genes that suppress myogenic differentiation, i.e., *Id1*, *Hes1*, and *Hey1*, are direct target genes of BMP6.
- BMP and Notch signaling share *Hes1* and *Hey1* as direct targets.

**Abstract:**

Muscle stem cells (MuSCs) are the cellular source for the generation and regeneration of skeletal muscle. Proper muscle growth requires precise control over the differentiation and self-renewal of MuSCs. Signaling systems, such as bone morphogenetic proteins (BMPs) and Notch, suppress the myogenic differentiation of MuSCs. This allows the expansion of the progenitor pool necessary for muscle growth. To better understand the molecular mechanisms and target genes of BMPs during myogenesis, we examined the response of adult mouse MuSCs to BMP6. BMP6 stimulation of freshly isolated MuSCs suppressed myogenic differentiation. Short-term stimulation (one hour) rapidly increased the expression of classical BMP target genes, such as *Id1*, as well as Notch pathway genes, including *Hes1*, *Hey1*, *Lfng*, and *Snai1*. We used Cleavage Under Targets and Tagmentation (CUT&Tag) to generate whole-genome binding profiles for pSMAD1/5/9 and SMAD4, which are transcriptional effectors of the BMP pathway. This method detected dynamic binding in promoters and regulatory elements of direct BMP targets, including Notch pathway genes. Our data demonstrate that BMP6 is a potent suppressor of MuSC differentiation and reveal that a subset of well-characterized anti-myogenic genes (i.e., *Hes1* and *Hey1*) are shared targets of the BMP and Notch pathways.

## 1. Introduction

Adult muscle stem cells (MuSCs), also known as satellite cells, play an important role in muscle homeostasis and regeneration. MuSCs either proliferate to expand the MuSC pool or differentiate to form new muscle fibers [1,2,3]. In healthy, uninjured skeletal muscle, MuSCs are quiescent and express the paired-box transcription factor *Pax7*. In response to physiological triggers, such as exercise, muscle injury, or disease, MuSCs proliferate and produce myogenic differentiation factors such as MYOD and MYF5, in addition to PAX7. Terminal differentiation and fusion into myofibers are accompanied by the upregulation of myogenin (*Myog*), a transcription factor, as well as fusion genes such as Myomaker (*Mymk*) [1,4,5,6]. This is followed by exit from the cell cycle.

MuSCs constantly respond to environmental triggers, such as a broad spectrum of signaling molecules, including bone morphogenetic proteins (BMPs), Notch ligands, hepatocyte growth factor (HGF), insulin-like growth factor 1 (IGF-1), transforming growth factor-β (TGF-β), fibroblast growth factors (FGFs), and Wnts (reviewed in [7]). These molecules initiate signaling cascades that control gene expression. In addition to receiving cues from other cell types, MuSCs communicate with each other via cell-to-cell interactions [8,9] and autocrine signaling [10]. The molecular mechanisms and cues that regulate MuSC function are the subject of intense research.

The BMP pathway is one of the signaling cascades that regulate MuSCs [11,12]. BMPs belong to the TGF-β superfamily and bind to type I and type II receptor serine/threonine kinases. Upon ligand binding, these kinases trans-phosphorylate the effector proteins SMAD1, SMAD5, and SMAD9 [13]. Phospho-SMAD1/5/9 form complexes with SMAD4 and translocate into the nucleus, where they modulate the transcription of BMP target genes, including members of the inhibitor of DNA binding (*Id*) gene family [14]. TGF-β, on the other hand, uses pSMAD2/3 and SMAD4 for signaling [13]. ID proteins strongly bind to the E proteins E12 and E47, which are heteromeric partners of the myogenic factors MYF5, MYOD1, and MYOG. Thus, ID proteins deplete myogenic factors of their partners required for optimal DNA binding by tethering E proteins away [15].

Disrupting BMP signaling during postnatal myogenesis severely impacts muscle formation [11]. Activation of the pathway by exogenous BMPs in culture prevents the differentiation of MuSCs and prolongs their proliferative phase [11,16,17]. Conversely, abrogating BMP signaling in vivo using genetic ablation of the BMP receptor type 1A (*Bmpr1a*) or inhibiting BMP signaling by overexpressing an inhibitor (*Noggin*) or an inhibitory SMAD (*Smad6*), promotes differentiation [11]. Thus, BMP signaling suppresses MuSC differentiation [16,17]. BMP inhibition also directly affects muscle fibers, resulting in muscle wasting [12,18].

The Notch signaling system represents an evolutionarily conserved pathway [19,20]. Signal-sending cells present membrane-bound ligands such as Delta-like (DLL1/3/4) or Jagged (JAG1/2) on their surface, while signal-receiving cells express the Notch receptors (NOTCH1–4) [21]. Upon ligand binding, the Notch receptor is cleaved by γ-secretase and the Notch intracellular domain (NICD) is released and translocates to the nucleus, where it binds to RBPJ [22]. The NICD–RBPJ complex then activates Notch target genes, among them the Hes/Hey family of transcriptional repressors [23].

Notch signaling is an important regulator of myogenesis, controlling the balance between the self-renewal and differentiation of MuSCs during development and regeneration [24,25,26]. Presentation of exogenous ligands stimulates Notch signaling, which suppresses differentiation. Conversely, pharmacological inhibition or mutations that interfere with Notch signaling promote differentiation [9,27]. Hes/Hey factors, which are induced by Notch signaling, inhibit differentiation in vivo and in cultured MuSCs by directly repressing *Myod1* and *Myog* transcription [27,28,29]. Interestingly, *Hes1*, *Myod1*, and *Dll1* are expressed in an oscillating manner in activated MuSCs [9,27]. Notch signaling plays an additional role in the adult muscle: maintaining MuSCs in a quiescent state [30,31]. In summary, activation of both the Notch and BMP pathways suppresses myogenic differentiation.

Previous investigations of BMP signaling in myogenesis have primarily focused on cellular consequences, such as differentiation, through the long-term exposure of MuSCs to BMPs [16,17]. Mechanistic studies have described crosstalk between the BMP and Notch pathways in myogenic cells, relying on classical techniques such as pull-down experiments and promoter assays [32,33].

Here we use RNA-sequencing (RNA-seq) and Cleavage Under Targets and Tagmentation (CUT&Tag) in MuSCs to characterize direct BMP target genes. This allows us to better understand the fine-tuning of differentiation by BMPs. Our analysis focuses on immediate and direct changes in gene expression, i.e., short-term responses to BMPs. MuSC stimulation with BMPs rapidly resulted in the upregulation of classical targets, such as *Id1*, as well as Notch pathway genes including *Hes1*, *Hey1*, *Lfng*, and *Snai1*. This upregulation occurred independently of Notch signaling. Additionally, our experiments revealed that transcription factors acting downstream of BMPs (i.e., pSMAD1/5/9 and SMAD4) bind to promoters and regulatory sequences of Notch pathway genes. These findings highlight a direct control of the Notch pathway by BMP signaling and provide a mechanistic basis for the effects of BMPs on MuSC differentiation.

## 2. Results

### 2.1. Rapid Activation of the BMP Signaling Cascade and Notch Genes in MuSCs upon BMP Stimulation

**Figure 1:**
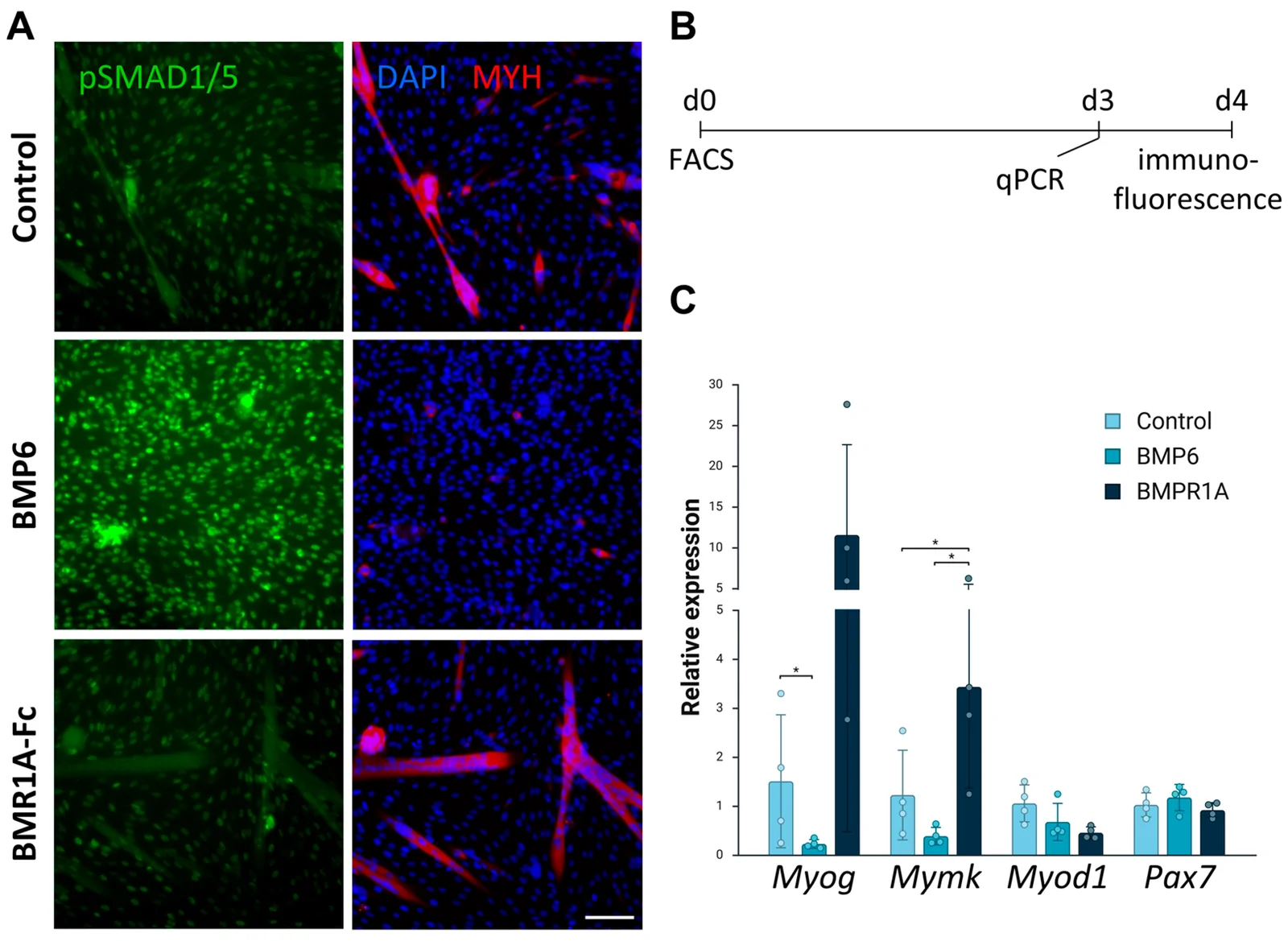
**Bone morphogenetic protein 6 (BMP6) inhibits the differentiation of muscle stem cells (MuSCs).** MuSCs were cultured in growth medium (control) or in growth medium containing BMP6 [100 ng/mL] or the antagonistic BMP receptor 1A fragment (BMPR1A-Fc) [200 ng/mL]. Their differentiation was analyzed by immunofluorescence and qPCR. (**A**) Immunofluorescence with anti-SMAD1/5 (left panels, green) or anti-myosin heavy chain (right panels, red) antibodies in day 4 cultures. DAPI was used as a counterstain (blue). Scale bar: 50 µm. (**B**) Schematic timeline of the experiment. (**C**) qPCR analysis of Myogenin (*Myog*), Myomaker (*Mymk*), *Myod1*, and *Pax7* on day 3. Note that *Myog* and *Mymk* expression is induced at the onset of terminal differentiation. Fold change (FC) calculations were performed using the 2^−ΔΔCt^ method [35]. The two-tailed paired *t*-test was used for statistical analyses. Significance level: *, *p* < 0.05. Values are shown as mean ± SD of *n* = 4 biological replicates per condition.

It has been reported that BMP signaling suppresses the myogenic differentiation of MuSCs. While most studies have used BMP4, other BMPs have been reported to possess similar activity [12,16,17,33,34]. Previously, we found that BMP6 is dominantly expressed in MuSCs [11]. Therefore, we tested its activity in an experiment in which MuSCs were exposed to BMP6 over a period of four days in culture. As a control, we used a cell culture medium without additional exogenous BMP6 (Figure 1B). BMPs are known to be present in serum [11,17]. To deplete BMPs in the medium, we used the soluble extracellular domain of the BMP receptor 1A (BMPR1A-Fc) as an additional control. Analysis of phospho-SMAD1/5 by immunofluorescence on day 4 demonstrated an enhanced signal intensity in the presence of BMP6 (Figure 1A). Myotube formation was observed beginning on day 4 of culture and was visualized and analyzed using a pan-myosin heavy chain antibody (Figure 1A). This showed that exogenous BMP6 strongly suppressed differentiation. In contrast, the addition of the soluble antagonistic BMP receptor enhanced differentiation. The fusion index in control cultures on day 4 was 16.8 [SD 5.7], was 5.7 [SD 2.3] in the presence of exogenous BMP6, and was 25.0 [SD 2.5] in the presence of the antagonistic BMP receptor. The expression of *Myog* and *Mymk* marked the onset of differentiation on day 3 (Figure 1C). Their expression was decreased with the addition of BMP6 and increased with the addition of the antagonistic BMP receptor fragment (Figure 1C). Thus, BMP6 was confirmed to be a potent inhibitor of differentiation in cultured MuSCs.

**Figure 2:**
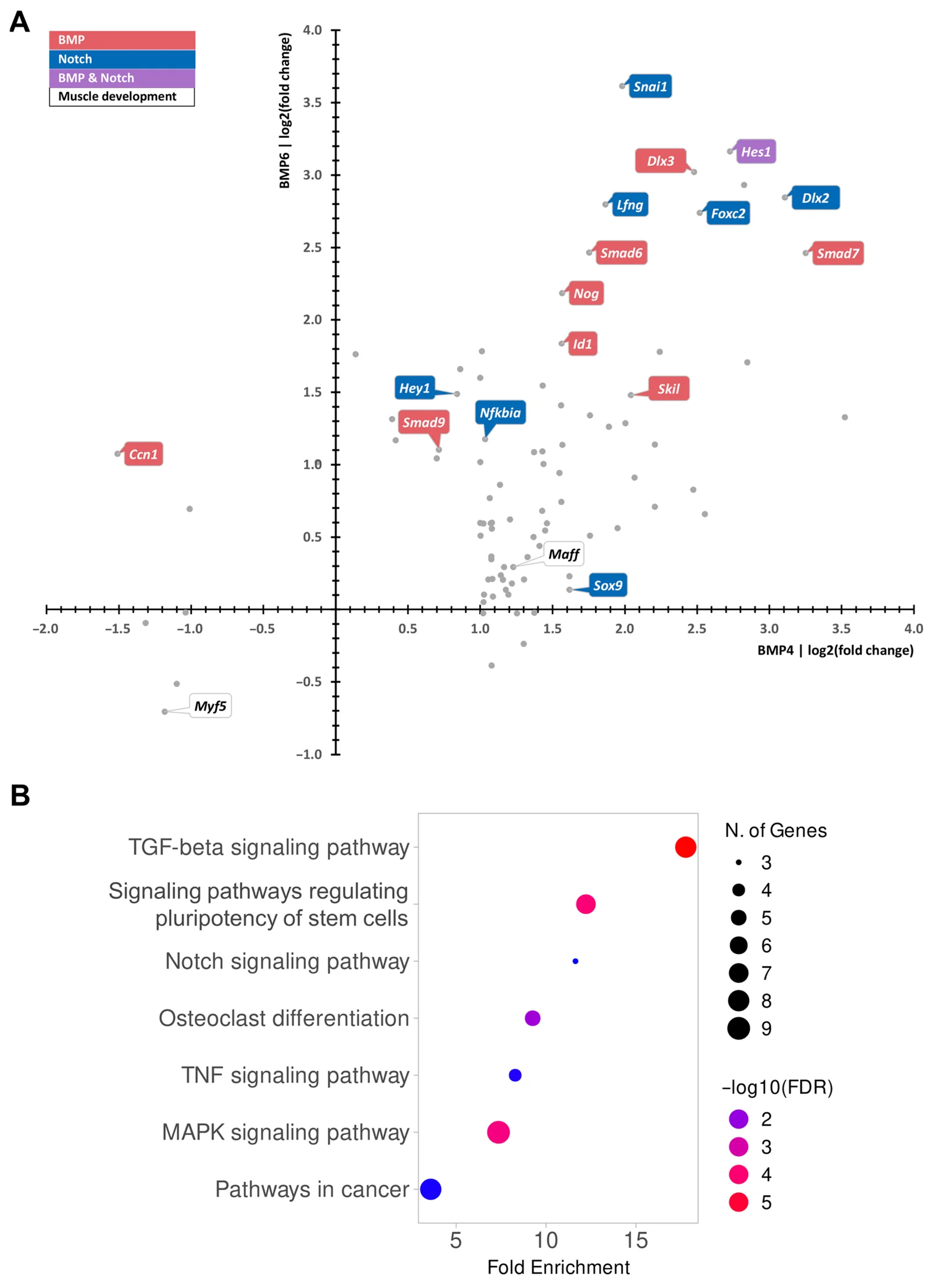
**Regulation of BMP and Notch pathway genes in BMP-stimulated, cultured mouse MuSCs.** (**A**) BMP4-regulated (X-axis) versus BMP6-regulated (Y-axis) genes revealed by RNA-sequencing (RNA-seq). All genes that exhibited an FC of ≥2.0 or ≤0.5 and a false discovery rate derived by Benjamini–Hochberg correction (FDR(BH)) of <0.1 after stimulation with BMP6 and/or BMP4 are represented by a grey dot. Genes of the BMP pathway and the Notch pathway are highlighted in color (BMP in red, Notch in blue, BMP & Notch in purple). Genes controlling skeletal muscle development are shown on a white background. (**B**) Dot plot showing significantly enriched signaling pathways (FDR < 0.05). The size of the dot reflects the number of pathway genes upregulated in response to BMP stimulation, and the color denotes the −log_10_FDR(BH); *n* = 2 biological replicates per condition.

Previous experiments analyzing the time course of MuSC responses to BMPs showed that expression of *Id1*, a direct BMP target gene, had already peaked 1 h after BMP4 exposure (Figure S1D in reference [11]). In order to systematically define the direct targets of BMP, we employed short-term stimulation and compared the responses of MuSCs to BMP6 and BMP4 using RNA-seq after 1 h of stimulation. Cells treated with an antagonistic BMP receptor were used as a control. This analysis revealed substantial overlap between BMP6- and BMP4-responsive genes (Figure 2). Using a stringent cut-off of a fold change (FC) of ≥2.0 or ≤0.5, we identified 89 differentially expressed genes (DEGs) that were controlled by BMP6 and/or BMP4. The false discovery rate was derived using the Benjamini–Hochberg correction (FDR(BH)) and set to <0.1, as suggested for exploratory experiments with a low number of biological replicates by Love et al. (2014) [36] (Figure 2A, Table S2; see Figure S1A for effects in individual samples). These 89 DEGs included sixteen genes from the BMP and Notch signaling pathways as well as two additional genes controlling skeletal muscle development (Figure 2A; see Table S1 for a full list of pathway genes). The majority of DEGs were found to be upregulated. Of the few downregulated genes, one encoded the myogenic determination factor 5 (*Myf5*). As we did not identify any pSMAD1/5/9 or SMAD4 binding sites upstream of *Myf5* (see Section 2.4), we hypothesize that *Myf5* downregulation might have been achieved indirectly through SNAI1 binding to its promoter, resulting in transcriptional repression, as was described in alveolar rhabdomyosarcoma cells [37]. In our data, *Snai1* expression was highly upregulated in response to BMP treatment.

Gene ontology analysis [38,39] of DEGs observed after BMP6/4 treatment demonstrated an enrichment of the terms “TGF-β signaling”, “signaling regulating pluripotency”, and “Notch signaling” (Figure 2B). Indeed, eight of the 89 genes regulated by BMP6/4 encode components of the Notch pathway. For example, *Hes1* and *Hey1*, which are important Notch effectors that repress the differentiation of MuSCs by suppressing the transcription of *Myod1* and *Myog* [40,41], were found to be upregulated in the RNA-seq experiment. Additionally, *Lfng*, a gene that encodes a glycosyltransferase that modifies the Notch receptors, and *Snai1*, a Notch pathway target that is known to modify MYOD function [42,43], were strongly upregulated by BMP6 and BMP4. The RNA-seq results were validated using mRNA expression arrays and qPCR arrays (Figures S1B and S2A–C; Table S3).

### 2.2. Activation of Notch Genes Is Independent of Notch Pathway Activity and Protein Synthesis

**Figure 3:**
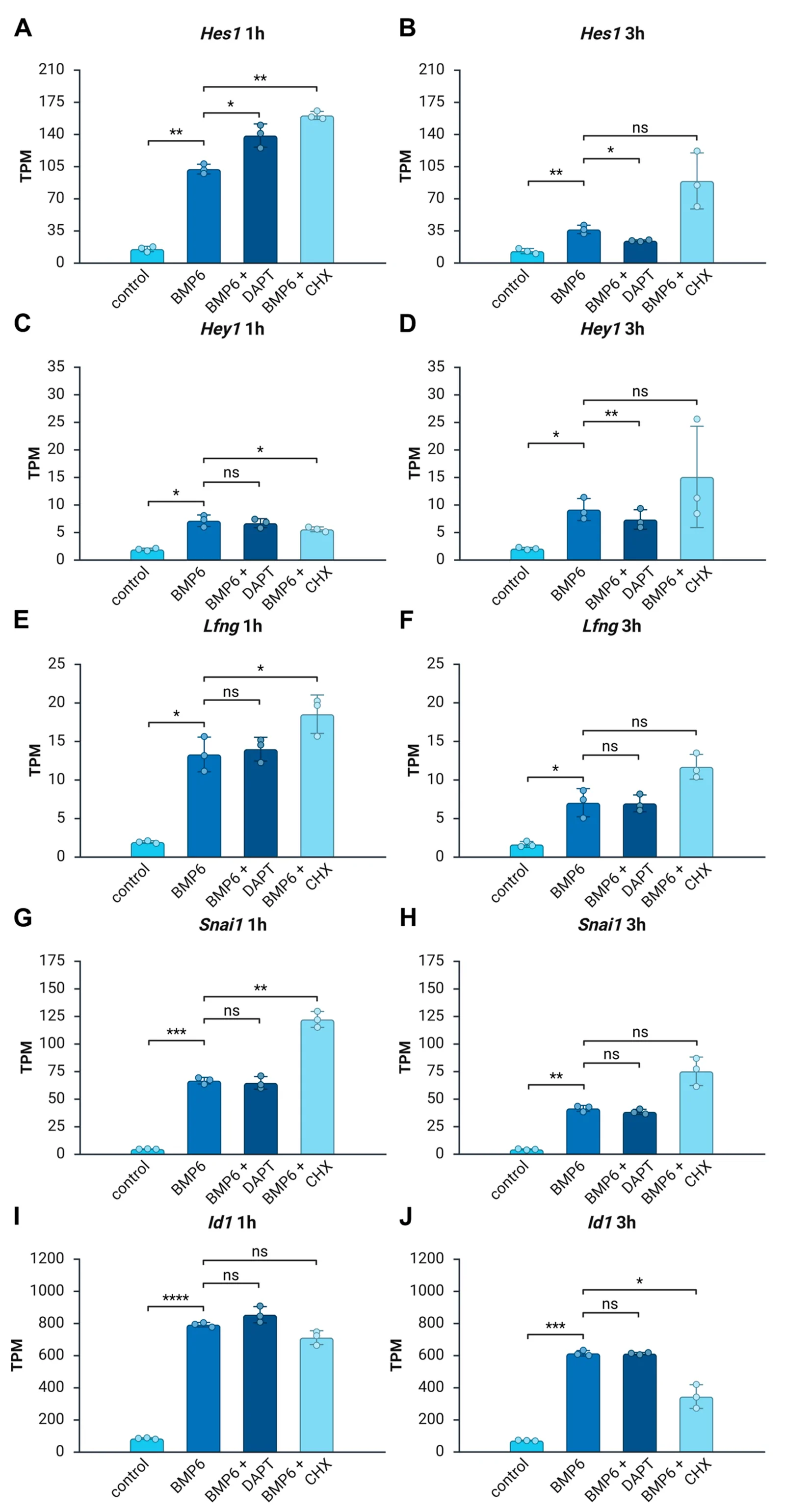
**Short-term effects of BMP6 on gene expression occur independently of Notch signaling.** Transcripts per million (TPMs) of selected BMP and Notch pathway genes induced with or without stimulation of MuSCs with BMP6 were determined by RNA-seq. Cells were starved for six hours in the presence of the antagonistic BMP receptor fragment BMPR1A-Fc and subsequently exposed for one and three hours to BMP6, BMP6 plus DAPT (Notch signaling inhibitor), or BMP6 plus cycloheximide (protein synthesis inhibitor). The panels depict analyses of the Notch pathway genes *Hes1*, *Hey1*, *Lfng*, and *Snai1* (**A**–**H**) and the classical BMP target inhibitor of DNA binding 1 (*Id1*) (**I**,**J**). The two-tailed paired *t*-test was used for statistical analyses. Significance levels: *, *p* < 0.05; **, *p* < 0.01; ***, *p* < 0.001; ****, *p* < 0.0001; ns, not significant. Values are shown as the mean ± SD of *n* = 3 biological replicates per condition.

The strong induction of Notch pathway genes such as *Hes1*/*Hey1* just 1 h after the addition of BMP6 to MuSCs suggests that BMP6 directly regulates the expression of these genes. To eliminate the possibility of other mechanisms, such as the induction of Notch signaling or the synthesis of new proteins, we examined the responses to BMP6 using RNA-seq in the presence or absence of **(i)** DAPT, a Notch signaling inhibitor, or **(ii)** cycloheximide, a protein synthesis inhibitor. DAPT had little to no effect on the BMP-induced expression of *Hes1*, *Hey1*, *Lfng*, and *Snai1* or on the expression of the known direct BMP target gene *Id1*, at 1 and 3 h of BMP treatment (Figure 3). Cycloheximide further enhanced the BMP6-dependent expression of *Hes1*, *Lfng*, and *Snai1*, an effect that appeared to be more pronounced after 3 h, suggesting the presence of negative feedback loops. Negative feedback loops have indeed been extensively studied in the context of Hes1, which transcriptionally represses its own promoter [44]. The time required for the transcription, splicing, and translation of *Hes1* was estimated to be around 40 min [45]. Therefore, the full establishment of the feedback loop requires time.

Aside from *Hes1*/*Hey1*, several direct target genes of the canonical Notch signaling pathway had been previously identified in MuSCs [28,46]. Among these, *Col5a1* and *Sgca* responded to BMP6, while the other 11 genes did not (Figure S3). The changes in *Col5a1* and *Sgca* transcript levels were smaller than a factor of 1.5 and were not reversed by Notch inhibition, further supporting the notion that BMP6 controls *Hes1*/*Hey1* in MuSCs independently of Notch signaling.

### 2.3. BMP6 Stimulation Induces the Production of HES1 Protein and Synchronizes Hes1 Oscillations

**Figure 4:**
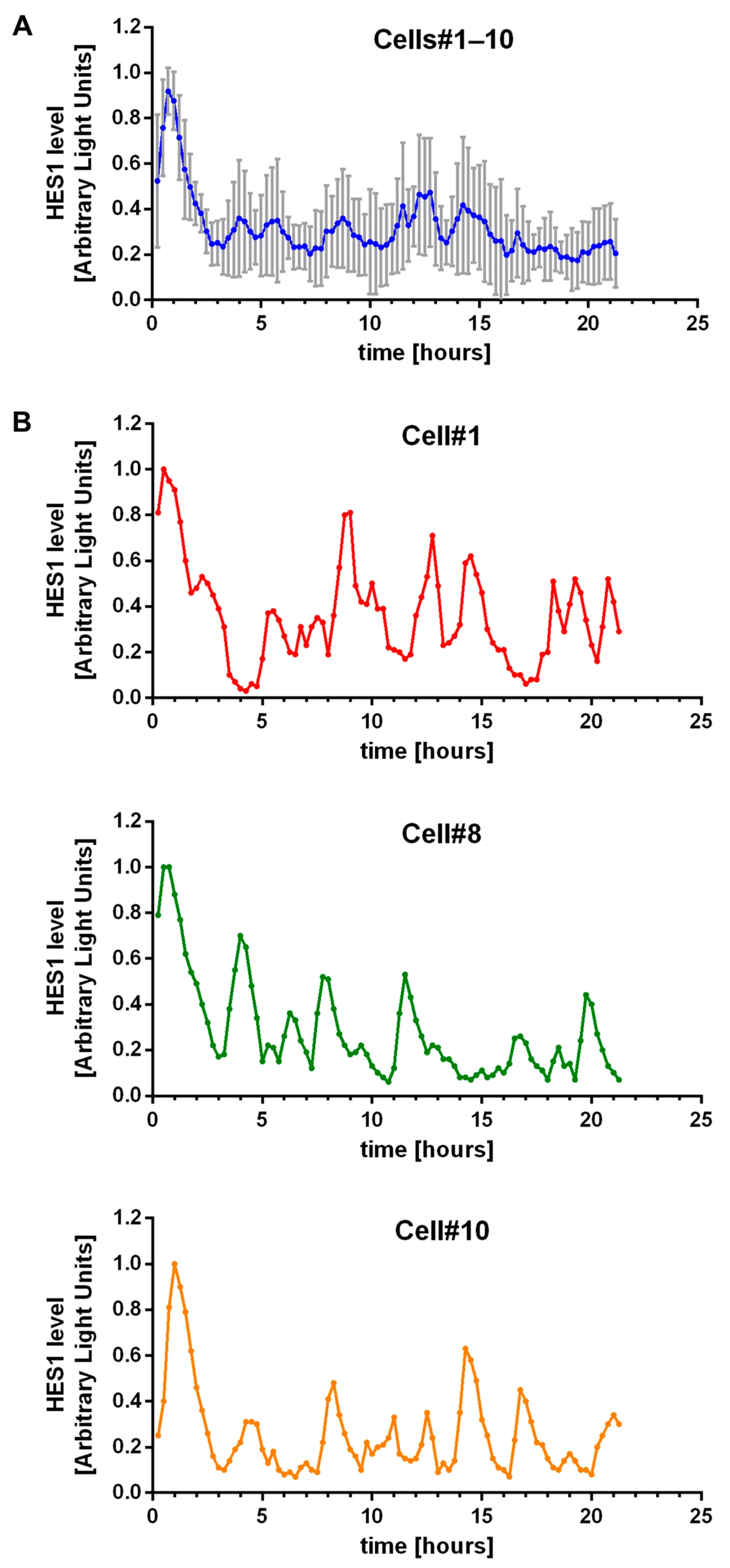
**Induction of HES1 protein by BMP6.** Luciferase activity produced in response to BMP6 in MuSCs of the Hes1-nLuc strain (luciferase coding sequences fused to the endogenous Hes1 locus; the HES1-nLuc fusion protein is produced by the gene). Live imaging of cells was used to record the luciferase signals over prolonged periods. (**A**) Average ± SD of luciferase signals obtained from a total of 10 individual cells. (**B**) Luciferase activity in response to BMP6 in three individual cells; see also Figure S4 for the remaining cells, each cell is depicted in a separate color. One of *n* = 5 biological replicates is shown.

Next, we investigated whether the HES1 protein was produced in response to BMP stimulation. To monitor this, we used MuSCs obtained from a *Hes1*-*nLuc* mouse strain, in which the luciferase coding sequences have been inserted into the endogenous *Hes1* gene. This results in the production of a HES1-nLuc fusion protein. After starvation, the cells were exposed to BMP6, and the luciferase activity was monitored using live imaging of single cells. The luciferase signals were then averaged over 10 cells (Figure 4A). This revealed rapid production of the HES1-nLuc protein, a peak in luciferase activity at 1 h after the addition of BMP, and a subsequent decline. Thus, BMP synchronously boosts the production of the HES1 protein in MuSCs. The subsequent reduction in luciferase activity likely reflects the onset of the negative feedback [44]. This reduces *Hes1* transcription due to repression by the HES1 protein. The short half-life of HES1 then allows a new round of *Hes1* expression to begin, resulting in oscillation. As oscillations are asynchronous and noisy, only intermediate levels of luminescence activity are then observed in the averaged traces (Figure 4A,B; see also [41]).

### 2.4. CUT&Tag Detects Direct Binding of BMP Pathway Effectors to Notch Genes

Next, we tested whether the Notch pathway genes that had been upregulated were bound by SMADs, which are the transcriptional BMP effectors. To do this, we performed CUT&Tag using antibodies against pSMAD1/5/9 and SMAD4, the transcription factors that cooperatively bind to and activate BMP target genes. Additionally, we performed CUT&Tag using an antibody against H3K27ac, a histone modification that marks active promoters and enhancers [47]. To correlate the CUT&Tag experiments with changes in gene expression, the same cells were used in a parallel RNA-seq experiment (Figures S1B and S6, Table S4).

**Figure 5:**
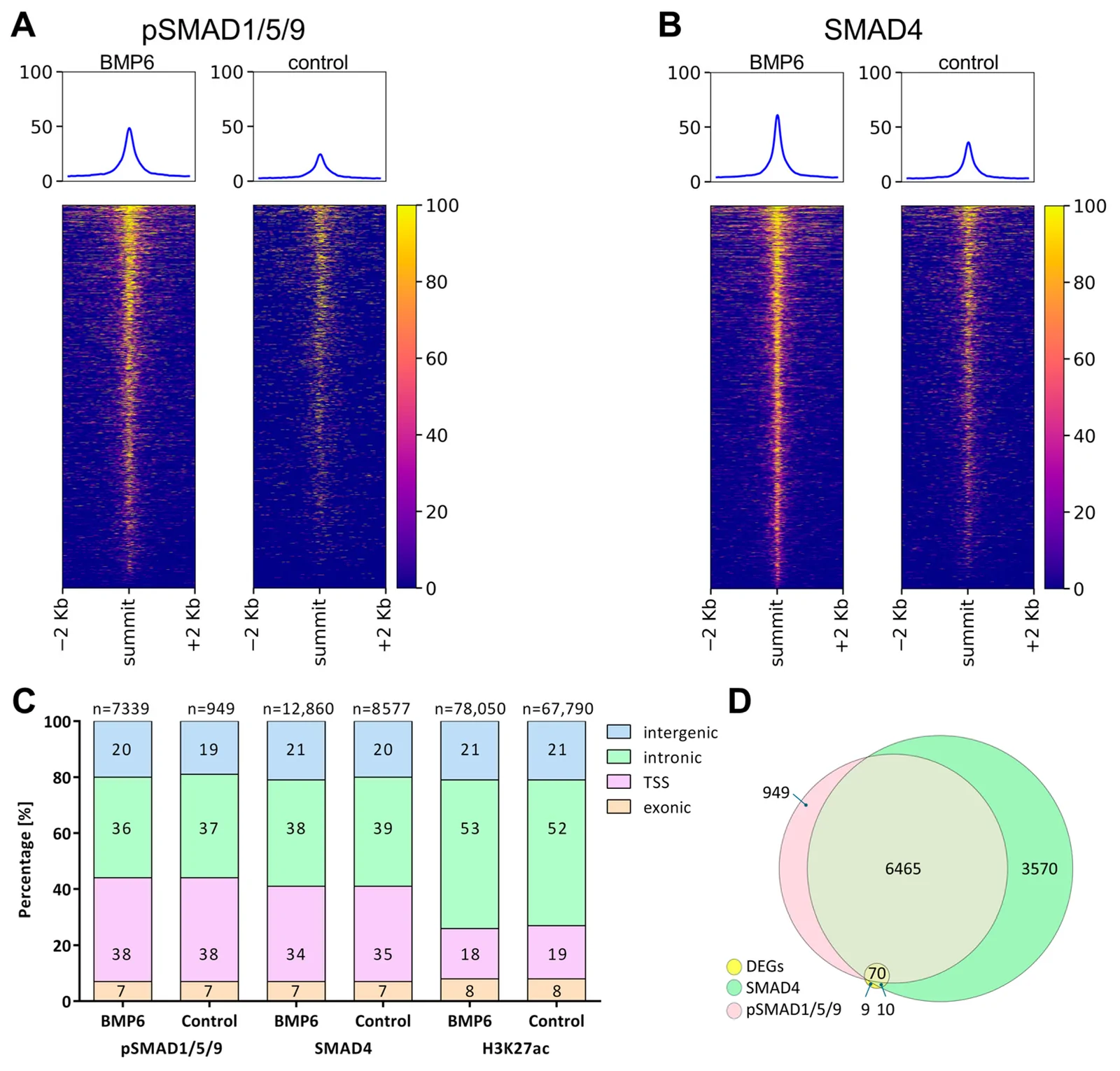
**Binding of pSMAD1/5/9 and SMAD4 to chromatin of MuSCs before and after BMP6 stimulation.** pSMAD1/5/9 and SMAD4 peaks determined by Cleavage Under Targets and Tagmentation (CUT&Tag) are enriched at transcription start sites (TSSs) and differentially expressed genes (DEGs). (**A**,**B**) Average profiles and heatmaps of pSMAD1/5/9 (**A**) and SMAD4 (**B**) peaks from BMP6-treated versus control cells centered around the peak summit. One of *n* = 2 biological replicates is shown. (**C**) Locations of SMAD and H3K27ac peaks in relation to the closest gene in BMP6-treated versus control cells. SMAD peaks are enriched in TSSs and intronic sequences. (**D**) Venn diagram demonstrating that the majority of DEGs are bound by pSMAD1/5/9 and/or SMAD4 in the gene body ±20 kb in BMP6-stimulated MuSCs.

In BMP6-stimulated cells, we detected 7339 pSMAD1/5/9 and 12,860 SMAD4 binding events compared to 949 and 8577 peaks, respectively, in control cells. Therefore, the changes in pSMAD1/5/9 binding were greater than those observed for SMAD4. It should be noted that TGF-β and BMP signaling converge on SMAD4. The smaller changes in SMAD4 binding might indicate that residual TGF-β signaling was present during the experiment. In summary, BMP6 treatment resulted in inducible SMAD binding, and not only the number of binding sites but also the average peak heights increased in response to BMP6 (Figure 5A,B and Figure S7A,B).

Most pSMAD1/5/9 and SMAD4 peaks were found in introns (~38%) or transcription start sites (TSSs; ~36%). The next most common locations were intergenic regions (~20%) and exons (7%). This distribution was similar in control and BMP6-stimulated cells (Figure 5C). However, H3K27ac peaks were found at higher proportions in intronic regions (~53%) and at lower proportions at TSSs (~19%; Figure 5C). Most pSMAD1/5/9 and SMAD4 peaks overlapped with H3K27ac peaks (range: 95.7–99.6%), indicating their location at active promoters and enhancers.

**Figure 6:**
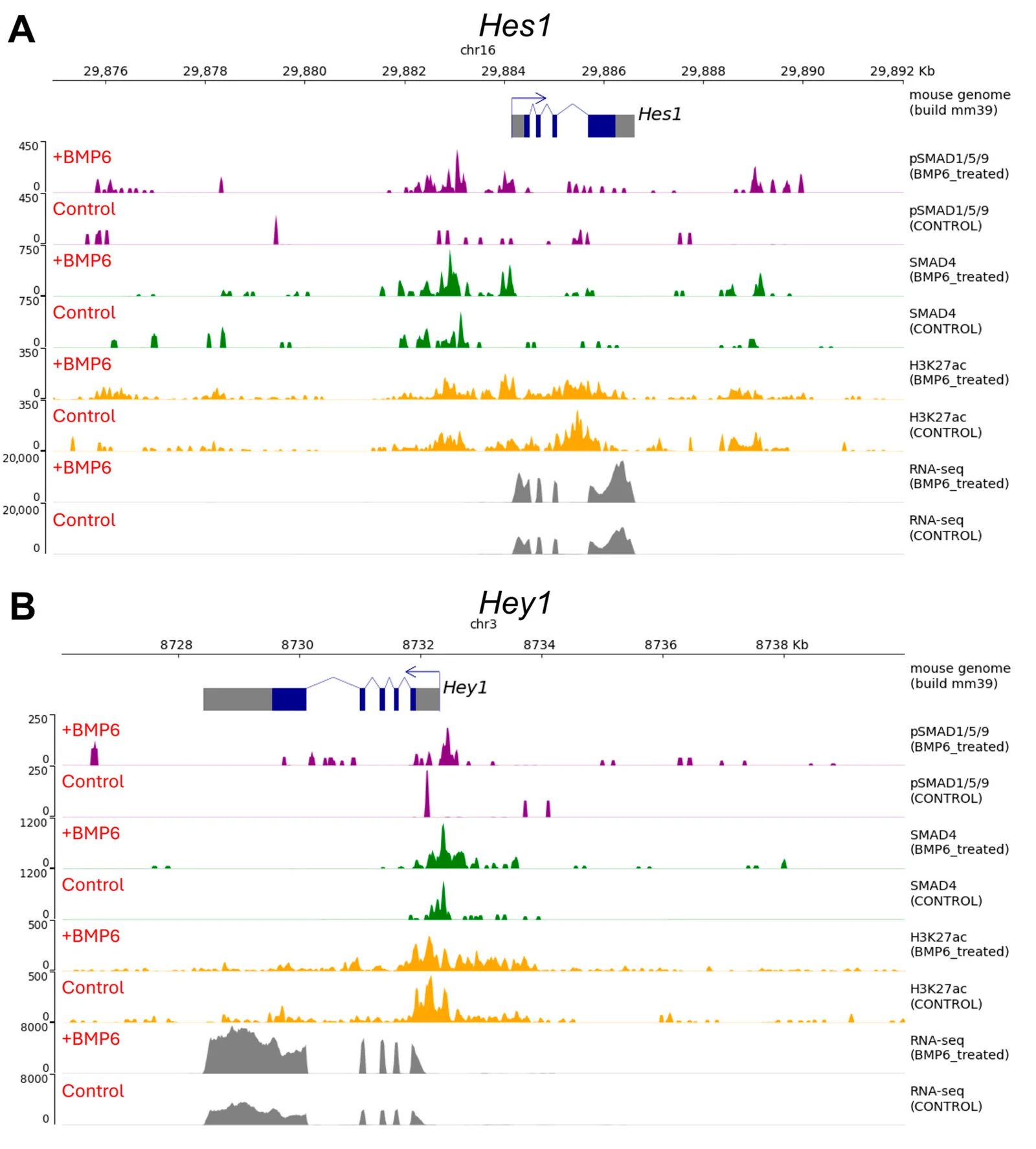
**Binding of SMADs to BMP-regulated genes.** (**A**,**B**) Binding of pSMAD1/5/9 and SMAD4 to the promoters of *Hes1* (**A**) and *Hey1* (**B**), encoding two central effector proteins of the Notch pathway. Shown are the CUT&Tag traces of pSMAD1/5/9 (BMP6 versus control) in purple, SMAD4 traces (BMP6 versus control) in green, as well as analyses of H3K27ac (BMP6 versus control) in orange. RNA-seq traces (BMP6 versus control) are depicted in grey at the bottom. One of *n* = 2 biological replicates is shown. See Figure S7 for examples of SMAD binding to additional loci.

Of the 89 DEGs, 80 were bound within ±20 kb of the gene body by pSMAD1/5/9 and/or SMAD4 after BMP6 stimulation (Figure 5D). Of these genes, 39 were identified as differentially bound between BMP-treated and control cells. For example, six genes from the BMP and Notch pathways were identified, including *Id1*, *Lfng*, and *Snai1* (Figure 6A,B and Figure S7C,D; Table S5). A visual inspection of the traces for the Notch effectors *Hes1* and *Hey1* revealed increased binding of pSMAD1/5/9 and SMAD4, though DiffBind and DESeq2 did not identify these genomic regions as differentially bound (Figure 6A,B).

The H3K27ac peaks associated with DEGs remained unchanged in the presence or absence of BMP6. This reflects the brief exposure to BMP6 and the stability of this histone mark. We attribute the observed changes in gene expression to variations in transcription factor binding, rather than to changes in the chromatin state. The data generated in the CUT&Tag and the associated RNA-seq experiments can be explored freely in a custom UCSC browser session via the following link: https://genome.ucsc.edu/s/mschue/mouse_Smad4_pSmad1 (accessed on 10 August 2026).

In conclusion, stimulating adult mouse MuSCs with BMP6 quickly changes the expression of not only BMP target genes but also Notch pathway genes, including *Hes1* and *Hey1*. After one hour of stimulation with BMP6/4, we detected a consensus set of genes that were differentially expressed. Our data suggest that these changes in gene expression are directly caused by the differential binding of pSMAD1/5/9 and SMAD4.

## 3. Discussion

In this study, we investigated the transcriptional response of cultured MuSCs to short-term BMP stimulation. Using RNA-seq, microarray, and qPCR array analyses, we detected rapid changes in gene expression in response to BMP exposure. CUT&Tag analyses using anti-pSMAD1/5/9 and anti-SMAD4 antibodies revealed the dynamic binding of these factors to promoters and regulatory elements of DEGs. A substantial proportion of the DEGs bound by SMADs encode components of the Notch signaling pathway. These include the well-characterized Notch effector genes *Hes1* and *Hey1*, which repress myogenic differentiation [29,41,48]. Our experiments consequently provide mechanistic insight by showing that a subset of anti-myogenic genes (*Hes1*, *Hey1*) are shared target genes of the BMP and Notch pathways.

Our CUT&Tag analysis revealed that pSMAD1/5/9 binding responded strongly to BMP6, while the response of SMAD4 binding was less pronounced. SMAD4 is known to be recruited to BMP and TGF-β target genes [49]. Therefore, the residual TGF-β present in our experiments may account for background SMAD4 binding and a blunted BMP6 response with respect to SMAD4 binding.

Here, we demonstrate that BMP6 stimulates the expression of several genes in the Notch pathway, including the Notch target genes *Hes1* and *Hey1*. Previous analyses have noted that *Hes1* and *Hey1* are induced by BMP [33,50] or TGF-β [32,51,52] in different cell types. These analyses relied on transfected *Hes1* and *Hey1* reporter constructs and forced NICD expression to mechanistically analyze the phenomenon. Some of these studies implicated NICD-mediated recruitment of SMAD1 or SMAD3 in regulating *Hes1*/*Hey1* expression [32,50]. Other authors have suggested that *Hey1* exhibits a biphasic response to TGF-β: a first phase dependent on SMAD3/SMAD4 recruitment but not Notch signaling and a second phase regulated by BMP-dependent production of a Notch ligand [52]. Our CUT&Tag data demonstrate that both pSMAD1/5/9 and SMAD4 bind to the *Hes1* and *Hey1* promoters after short-term BMP stimulation. These results support the idea that *Hes1*/*Hey1* transcription is directly stimulated through the canonical BMP signaling pathway, i.e., by recruiting pSMAD1/5/9-SMAD4 heteromers to *Hes1*/*Hey1* promoters and regulatory elements. Consistent with this idea, none of the Notch ligands was regulated by BMP signaling after short-term stimulation by BMP6. Furthermore, inhibitor experiments revealed that the upregulation of *Hes1*/*Hey1* expression does not depend on Notch signaling or active protein synthesis. However, cooperative binding and indirect recruitment of transcription factors are common mechanisms [53] that may additionally contribute to the BMP-dependent control of *Hes1*/*Hey1*.

The Hes/Hey family of transcriptional repressors [54] comprises important effectors of the Notch pathway in MuSCs, which suppress myogenic differentiation by directly repressing *Myod1* and *Myog* transcription [29]. Interestingly, *Hes1* transcription is tightly controlled by a feedback loop resulting in its oscillatory expression [9,27,41,44] and the repression of *Lfng*, a glycosyltransferase that modifies the Notch receptors [55]. Despite this negative feedback loop, *Hes1* was induced at high levels (i.e., by 4–5-fold) by short-term BMP treatment. This indicates that the feedback loop was not yet functional after short-term induction, likely due to delays in *Hes1* auto-inhibition caused by splicing and translation [41,45]. Similarly, *Id1* is strongly induced at this early time point. However, the prevention of DNA binding by MYOD1 through ID1-dependent tethering of E12 and E47 does not appear to have occurred yet, as MYOD targets such as *Des* and *Ckm* are not differentially regulated at this early time point. The short-term stimulation protocol combined with state-of-the-art technologies such as RNA-seq and CUT&Tag allowed us to identify direct BMP target genes. This demonstrated that the anti-myogenic genes *Hes1* and *Hey1* are shared target genes of the BMP and Notch pathways.

In conclusion, BMP6 suppresses myogenic differentiation. Our data indicate that BMP treatment represses myogenesis through indirect mechanisms such as controlling *Id1* and *Hes1*/*Hey1* expression. These factors work together to suppress muscle differentiation.

## 4. Materials and Methods

### 4.1. Animals and Isolation of MuSCs

MuSCs were isolated from adult mice aged between 4 and 20 weeks using fluorescence-activated cell sorting (FACS) or magnetic cell separation (MACS^®^) after euthanasia by cervical dislocation [11,28,56]. In a subset of experiments, mice carrying the Pax7-ZsGreen [57] (RRID:MGI:5829449) or Pax7-EGFP [58] (RRID:IMSR_JAX:036759) transgenes were used to isolate the Pax7^+^ MuSCs by FACS. Mice were fed ad libitum and held in specific-pathogen-free conditions on a 12 h dark–light cycle. All animal experiments were registered, performed in accordance with institutional guidelines for the care and use of laboratory animals, and approved by the local authorities (LaGeSo Berlin, Germany, T0183/15, approved on 10 July 2017, and X9008/20, approved on 18 September 2020).

### 4.2. Culture of MuSCs for Short-Term Stimulation Experiments

Cells were seeded in plating medium containing DMEM (Gibco, Darmstadt, Germany, 41966029), 15% fetal bovine serum (FBS; Gibco), 2.5 ng/mL basic FGF (Sigma, Taufkirchen, Germany, F3133), 10 ng/mL leukemia inhibitory factor (BioChrom, Berlin, Germany, W1655950010/Sigma, ESG1106), and 1% Pen/Strep (Gibco) into growth factor-reduced Matrigel^®^ (Corning, Wiesbaden, Germany, 356231)-coated dishes or plates and maintained in a humidified hypoxic atmosphere containing 3% O_2_ and 5% CO_2_ at 37 °C. After 24 h, 1× B27 without vitamin A (Gibco, 12587010) was added to the plating medium, and the MuSCs were cultured for another 24 to 48 h.

### 4.3. Short-Term Stimulation of MuSCs with BMPs and Treatment with Inhibitors

Prior to stimulation, the cells were starved in serum-free medium supplemented with 200 ng/mL soluble BMPR1A (recombinant mouse BMPR1A-Fc chimera, R&D Systems, Wiesbaden, Germany, 437-MR) and 1× B27 without vitamin A (Gibco, 12587010) for six hours. The cells were then stimulated with 100 ng/mL BMP6 (R&D Systems, 507-BP; for RNA-seq, CUT&Tag, inhibitor experiments, microarrays) or BMP4 (R&D Systems, 314-BP; for RNA-seq 1, pathway arrays) in serum-free medium for 1 or 3 h. The solution used to reconstitute the BMPs (4 mM HCl containing 0.1% BSA) served as a control in all experiments. For inhibition of Notch signaling, the γ-secretase inhibitor DAPT (MedChemExpress, Monmouth Junction, NJ, USA, HY-13027) was added to 20 µM 10 min before the start of the stimulation. For inhibition of protein synthesis, cycloheximide (Sigma, C1988) was added to 10 µM 10 min before the start of the stimulation.

### 4.4. Culture and Stimulation of MuSCs for the Long-Term Experiment

For the long-term stimulation experiments, cells were seeded in DMEM (Gibco, 31966021) containing 20% FBS (Gibco), 2.5 ng/mL basic FGF (Sigma, F3133), 10 ng/mL leukemia inhibitory factor (Sigma, ESG1106), and 1% Pen/Strep (Gibco) into growth factor-reduced Matrigel^®^ (Corning, 356231)-coated plates and maintained in a humidified hypoxic atmosphere of 3% O_2_ and 5% CO_2_ at 37 °C. After one day, 100 ng/mL BMP6 (R&D Systems, 507-BP), 200 ng/mL soluble BMPR1A (recombinant mouse BMPR1A-Fc chimera, R&D Systems, 437-MR), or 4 mM HCl containing 0.1% BSA was added, and MuSCs were cultured until day 3 or day 4.

### 4.5. HES1-Luc Experiment

For the HES1-nLuc experiments, cells were seeded on growth factor-reduced Matrigel^®^ (Corning, 354230) and expanded for 4 to 7 days in growth medium containing DMEM-F12 (Gibco, 11320033), 15% Fetal Calf Serum (Sigma, F7524), 10% Horse Serum (Gibco, 16050122), 1% Pen/Strep (Gibco, 15140122), 1% GlutaMAX supplement (Gibco, 35050061), 2.5 ng/mL basic FGF (Sigma, F3133), and 10 ng/mL LIF (Sigma, ESG1106) under normoxic conditions. Cells were passaged, seeded on Matrigel^®^-coated 35 mm glass-bottom dishes (MatTek, Ashland, MA, USA), and starved for 6 h in medium containing DMEM-F12, 2.5 ng/mL basic FGF, 10 ng/mL LIF, and 200 ng/mL soluble BMPR1A (recombinant mouse BMPR1A-Fc chimera, R&D Systems, 437-MR). One hour prior to stimulation, 1× NanoLuc substrate Endurazin (Promega, Walldorf, Germany, N257B) was added to allow equilibration. Cells were stimulated by replacing the medium with fresh growth medium supplemented with 100 ng/mL BMP6 (R&D Systems, 507-BP) and 1× Endurazin. Live-cell bioluminescence imaging was initiated as described previously [9]. Single frames were acquired every 15 min. To calculate and remove the background signal, the intensity of 10 spots on different frames was measured for each series.

### 4.6. RNA Isolation

For RNA-seq 1, the microarrays, and the pathway arrays, RNA was isolated from fresh MuSCs using the RNeasy Micro Kit (Qiagen, Hilden, Germany, 24004) according to the manufacturer’s instructions. Residual DNA was removed using the RNase-Free DNase Set (Qiagen, 79254). RNA quantity and quality were assessed using a NanoDrop^®^ 2000c spectrophotometer (Thermo Fisher Scientific, Dreieich, Germany, RRID:SCR_020309).

For the remaining experiments, MuSCs were trypsinized and frozen at −80 °C prior to RNA isolation. In the inhibitor experiment, total RNA was extracted using the NucleoSpin^®^ RNA kit (Macherey-Nagel, Düren, Germany, 740955) according to the manufacturer’s instructions. For RNA-seq 2, total RNA was extracted using the NucleoSpin^®^ RNA XS kit (Macherey-Nagel, 740902) according to the manufacturer’s instructions. RNA quantity and quality were assessed using a NanoDrop^®^ 2000c spectrophotometer (Thermo Fisher Scientific, RRID:SCR_020309).

### 4.7. RNA-Seq

For RNA-seq experiments, high RNA integrity corresponding to an RNA integrity number (RIN) of >8 was confirmed using the BioAnalyzer 2100 (Agilent Technologies, Waldbronn, Germany, RRID:SCR_019715) and the RNA 6000 Nano Kit (Agilent Technologies, 5067-1511) or the RNA 6000 Pico kit (Agilent Technologies, 5067-1513). Stranded libraries were prepared for RNA-seq 1 with the SureSelect strand-specific RNA library prep for Illumina multiplexed sequencing kit (Illumina, Berlin, Germany), quality-checked, and sequenced in single-end 50 bp mode to more than 40 million reads per sample with the Illumina HiSeq 4000 machine (Illumina, RRID:SCR_016386; performed by BGI Genomics, Shenzhen, China, RRID:SCR_024848).

For the RNA-seq 2 experiment, unstranded libraries were prepared using the Smart-seq2 protocol until the PCR preamplification of the cDNA [59] and continued with the Nextera XT DNA library prep kit (Illumina, FC-131-1096) using the MGI universal and index primers for PCR amplification. The libraries were quality-checked and sequenced in paired-end 100 bp mode to more than 60 million reads per sample with the DNBSEQ-G400 machine (RRID:SCR_017980; performed by BGI Genomics, RRID:SCR_024848).

For RNA-seq of the inhibitor experiments, stranded libraries were prepared according to BGI Genomics’ “DNBSEQ Eukaryotic Strand-specific Transcriptome Resequencing” protocol, quality-checked, and sequenced in paired-end 150 bp mode to more than 55 million reads per sample with the DNBSEQ-G400 machine (RRID:SCR_017980; performed by BGI Genomics, RRID:SCR_024848).

### 4.8. Bioinformatic Analysis of Transcript Abundance

Reads were aligned to the mouse reference assembly GENCODE M32 (GRCm39) using STAR v2.7.11a (RRID:SCR_004463) [60]. The resulting *.sam files were converted to *.bam files and then sorted and indexed using SAMtools v1.17 (RRID:SCR_002105) [61]. The sorted *.bam files were passed to StringTie v2.2.1 (RRID:SCR_016323) [62]. The resulting *.gtf files were merged using stringtie merge, and normalized read counts (transcripts per million (TPM) and fragments per kilobase of transcript per million mapped reads (FPKM)) were generated using stringtie ballgown. For differential expression analyses of RNA-seq 1 and 2, StringTie ballgown outputs were converted to raw counts using the prepDE Python script [62]. The DESeq2 v3 R module (RRID:SCR_015687) [36] was then run with default parameters to determine fold changes and FDR(BH). A fold change of ≥2.0 or ≤0.5 between stimulated and control samples and an FDR(BH) of <0.1 was used as a cut-off to define DEGs. Genes encoding long non-coding RNAs, miRNAs, antisense transcripts, and pseudogenes, i.e., genes starting with “Gm” or “Mir” or ending in “Rik”, “os” plus an optional number, or “-ps” plus an optional number, were not used for subsequent analyses. Merged lists from StringTie without an assigned gene name were also excluded.

Heatmaps for regulated genes were calculated from the raw fragment counts (RNA-seq) or raw intensity scans (microarrays) using the standard iDEP v2.4.4 pipeline with a paired (treated versus control) design and default settings [63]. Venn diagrams were created with eulerr.co (accessed 26 February 2026) [64]. The two-tailed paired *t*-test was used for statistical analysis of the inhibitor experiment in BioRender Graph using R 4.2.2. Raw and processed RNA-seq files are available on the Gene Expression Omnibus (GEO, RRID:SCR_005012) database under the accession numbers GSE159594 (for RNA-seq 1), GSE305746 (for RNA-seq 2), and GSE339586 (for the inhibitor experiments, RNA-seq 3).

### 4.9. Enrichment and Pathway Analysis

BMP signaling pathway (GO:0030509), Notch signaling pathway (GO:0007219), and skeletal muscle tissue development (GO:0007519) gene lists from AmiGO 2 (RRID:SCR_002143) v2.5.17 (from 13 October 2025) were displayed with “regulates”, filtered for mouse genes, and exported [38,39,65]. From the resulting gene lists, we removed complexes whose individual members were included in the same pathway list and corrected the spelling of human genes, often prefixed with “m”, to the consensus mouse gene names (see resulting lists in Table S1).

Differentially expressed genes detected by RNA-seq 1 were used for pathway analyses based on KEGG (RRID:SCR_012773) [66] pathways using ShinyGO v0.85 (RRID:SCR_019213) [67] with the settings of an FDR cutoff of 0.05 and a pathway size of 2 to 5000.

### 4.10. CUT&Tag Analysis of SMAD Binding

CUT&Tag was performed on 100,000 fixed, frozen cells per condition as described [68,69]. Briefly, the cells were fixed in 0.1% formaldehyde for 2 min, followed by quenching with glycine and slowly freezing with 10% DMSO. Cells were bound to beads and incubated with primary antibodies against H3K27ac (Abcam, Cambridge, UK, ab4729, RRID:AB_2118291), pSMAD1/5/9 (Cell Signalling, Danvers, MA, USA, 13820, RRID:AB_2493181), and SMAD4 (Cell Signalling, 38454, RRID:AB_2728776) at 4 °C overnight. On the next day, guinea pig anti-rabbit secondary antibody (Antibodies online, ABIN101961, RRID:AB_10775589) was added for one hour at room temperature. Subsequently, the cells were incubated with CUTANA pAG-Tn5 enzyme (EpiCypher, Durham, NC, USA, 15-1017) for 1 h at room temperature, followed by tagmentation for 1 h at 37 °C. Cleaved fragments were released before the DNA was extracted, precipitated, and amplified using 1× NEBNext^®^ High-Fidelity 2X PCR Master Mix (New England Biolabs, Frankfurt am Main, Germany, M0541L) with 5 µM barcoded primers (from [70]) in the Biometra TRIO PCR system (Analytik Jena, Jena Germany, RRID:SCR_027344). The libraries were cleaned up using Agencourt AMPure XP beads (Beckman Coulter, Krefeld, Germany, A63881), quality tested on the Bioanalyzer 2100 (Agilent Technologies, RRID:SCR_019715) using the Agilent High Sensitivity DNA Kit (Agilent Technologies, 5067-4626), and sequenced in paired-end 50 bp mode to more than 10 million reads per sample on a 10B lane of the Illumina NovaSeq X Plus (Illumina, RRID:SCR_024568).

### 4.11. Processing and Analysis of the CUT&Tag Data

Paired-end sequencing reads were subsampled to an equal total depth per antibody. Reads were then aligned to the mouse reference genome (GRCm39) using Bowtie2 version 2.5.4 (RRID:SCR_016368) [71] with the following parameters: --very-sensitive --no-mixed --no-discordant. Aligned reads were subsequently filtered using SAMtools version 1.21 (RRID:SCR_002105) [61] with the options -b -h -f 2 -F 256 -F 2048 -F 1024 -q 30 to retain only properly paired reads (mapping quality ≥ 30) aligned to autosomes and sex chromosomes while excluding reads mapped to ENCODE blacklisted regions. The filtered BAM files were converted to BED format using BEDTools version 2.31.1 (RRID:SCR_006646) [72].

Peak calling was performed independently for each biological replicate using JAMM version 1.0.7.6 (RRID:SCR_017049) [73] with the -r window parameter. To establish consensus peak sets, per-replicate peaks were subjected to Irreproducible Discovery Rate (IDR) analysis using the IDR package version 2.0.3 (RRID:SCR_017237) [74] with the parameters --rank signal.value --idr-threshold 0.05. IDR was run on the “filtered peaks” output from JAMM for transcription factors and on the “all peaks” output for the H3K27ac modification. Only peaks meeting the IDR threshold of 0.05 were retained when generating the consensus peak lists for each experimental condition.

Differential binding analysis between BMP6 treatment and control was conducted using DiffBind version 3.18.0 (RRID:SCR_012918) [75] in conjunction with DESeq2 version 1.48.1 (RRID:SCR_015687) [36] using the default parameters. Significantly differentially bound peaks of 400 bp in length were identified using an FDR(BH) threshold of 0.05. To associate peaks with DEGs from RNA-seq 1, differential peaks were intersected with candidate proximal and distal regulatory regions. Proximal regions were defined as −1.5 kb and +0.5 kb from the TSS of the RefSeq select isoform according to annotation GCF_000001635.27 and intersected with differential peaks using bedtools intersect -wo -a [peak_file] -b [TSS_file]. Distal regions were defined as 20 kb upstream of the 5′ UTR to 20 kb downstream of the 3′ UTR of the RefSeq select isoform (annotation GCF_000001635.27).

Profile plots and heatmaps of pSMAD1/5/9 and SMAD4 peaks were generated as follows. Normalized bigWig files were produced using the bamCoverage tool from deepTools version 3.5.5 (RRID:SCR_016366) [76] with the parameters --normalizeUsing RPKM --binSize 50. Binding score matrices spanning 2 kb upstream and downstream of the summit of all IDRed peaks were then computed using the computeMatrix reference-point command in deepTools with default parameters. Profile plots and heatmaps were generated from these matrices using the matplotlib package version 3.10.1 in Python 3.

For peak annotations, IDRed peaks were first assessed for overlap with regions spanning 2 kb upstream and downstream of the TSS of any RefSeq select isoform (annotation GCF_000001635.27) and annotated accordingly. In the absence of a TSS overlap, peaks were subsequently assessed for overlap with exonic regions, followed by intronic regions. Peaks that did not overlap any of the above features were classified as intergenic.

To determine the number of genes bound by each transcription factor in the BMP6-treated samples, a gene was counted as bound if at least one IDRed pSMAD1/5/9 or SMAD4 peak overlapped the distal region as defined above. Venn diagrams were created with eulerr.co (accessed 26 February 2026) [64].

To display the read piles and peak calls in a custom UCSC genome browser session, the files were loaded as custom tracks. For this purpose, all *.narrowPeak files were renamed to *.bed, and the column headers were deleted if present. All BED files were converted to bedGraph and then using the UCSC bedGraphToBigWig tool further to BigWig files [77].

Raw and processed CUT&Tag-seq files are available on the GEO database (RRID:SCR_005012) under the accession number GSE305745. The resulting BigWig files can be accessed at https://doi.org/10.5281/zenodo.21389765 (accessed on 10 August 2026).

### 4.12. Microarray Experiments and Their Bioinformatic Analysis

Total RNA from BMP6-treated and control cells was extracted and DNase-treated as described above. A RIN of >9 was confirmed using the Bioanalyzer 2100 (Agilent Technologies, RRID:SCR_019715) and the RNA 6000 Nano Kit (Agilent Technologies, 5067-1511). cDNA was synthesized using the GeneChip Whole Transcript (WT) Expression Plus Kit (Applied Biosystems, Darmstadt, Germany, 902280), followed by fragmentation and end-labeling using the GeneChip WT Terminal Labeling Kit (Applied Biosystems, 900670). The labeled cDNA was hybridized onto the Mouse Gene (MG) 1.0 ST Array (Applied Biosystems, 901169) at 45 °C for 16 h. After hybridization, the arrays were washed on the Affymetrix GeneChip 450 Fluidics Station Microarray Processor (Applied Biosystems, 00-0079, RRID:SCR_018034) and scanned with the GeneChip 3000 7G Microarray Scanner (Applied Biosystems, 00-0210, RRID:SCR_019341). The Mouse Gene 1.0 ST Array contains 750,000 unique 25-mer oligonucleotide probes interrogating 28,853 murine genes, with an average of 27 probes across the entire length of each gene.

Microarray data were analyzed with the R packages affy (RRID:SCR_012835) [78], oligo (RRID:SCR_015729) [79], and limma (RRID:SCR_010943) [80] of the Bioconductor suite (RRID:SCR_006442) [81]. Data quality control was carried out by creating pseudo images for each array using the oligo package and visually inspecting them for the presence of artifacts. The raw count *.CEL files were normalized using Robust Multichip Averaging (RMA) with quantile normalization, background correction, and annotation (using the MG 1.0 ST Chip Description File “mogene10stv1cdf” from Bioconductor). For downstream analyses, probes without gene annotations and lowly expressed genes (log_2_(normalized intensity) <5 in >50% of the samples) were removed. Different splice isoforms of the same gene were then summarized by keeping only the median of their intensities. Differential gene expression, fold changes, and FDR(BH) were calculated using the limma package. Raw and processed microarray files are available in the GEO database (RRID:SCR_005012) under the accession number GSE127798.

### 4.13. Pathway-Focused PCR Arrays

Total RNA from BMP4-treated and control cells was extracted and DNase-treated as described above. cDNA was synthesized using the RT^2^ First Strand Kit (Qiagen, 330404) and mixed with RT^2^ SYBR Green ROX™ qPCR Mastermix (Qiagen, 330523). Into each well of the RT^2^ Profiler™ PCR Array Mouse TGF-β/BMP Signaling Pathway (Qiagen, PAMM-035ZA, 330231) and the RT^2^ Profiler™ PCR Array Mouse Notch Signaling Pathway (Qiagen, PAMM-059ZA, 330231), 25 μL of this mixture was aliquoted. Real-time qPCR was run with the ABI PRISM 7000 Sequence Detection System (Applied Biosystems, RRID:SCR_021899). FC calculations between BMP-treated and control samples were performed using the 2^−ΔΔCt^ method [35]. As part of the analysis, C_t_ values were calculated in relation to the arithmetic mean of the five housekeeping genes *Actb*, *B2m*, *Gapdh*, *Gusb*, and *Hsp90ab1*. The paired two-tailed *t*-test was used for statistical analysis of the resulting FCs in GraphPad Prism 10. Calculated *p*-values were corrected for multiple comparison testing using an online FDR calculator (https://www.sdmproject.com/utilities/?show=FDR, accessed 18 July 2026). The level of significance was set to FDR(BH) < 0.1.

### 4.14. Real-Time Quantitative PCR

Total RNA from BMP6-treated, BMPR1A-treated, and control cells from day 3 of the long-term stimulation experiment was extracted and DNase-treated as described above. cDNA synthesis was performed using the PrimeScript RT Reagent Kit with gDNA Eraser (Perfect Real Time; Takara Bio, Saint-Germain-en-Laye, France, RR047) according to the manufacturer’s instructions. Real-time qPCR was performed with the StepOnePlus Real-Time PCR System (Applied Biosystems, RRID:SCR_015805) and the Biozym Blue S’Green qPCR Kit (Biozym, Hessisch Oldendorf, Germany, 331416S). The following oligonucleotide sets were used: *Myog* forward: 5′-CAG TAC ATT GAG CGC CTA CAG-3′, *Myog* reverse: 5′-GGA CCG AAC TCC AGT GCA T-3′, *Mymk* forward: 5′-CCG TGA CAT TCT GGA GTA CTT C-3′, *Mymk* reverse: 5′-CAT TGT GAA GGT CGA TCT CTG G-3′, *Myod1* forward: 5′-TAC AGT GGC GAC TCA GAT GC-3′, *Myod1* reverse: 5′-TAG TAG GCG GTG TCG TAG CC-3′, *Pax7* forward: 5′-AGC AAT GGC CTG TCT CCT C-3′, *Pax7* reverse: 5′-ACG TGG GCA AGC TGT CTC CTG-3′, *Gapdh* forward: 5′-GCA TGG CCT TCC GTG TTC-3′, and *Gapdh* reverse: 5′-GGG TGG TCC AGG GTT TCT TAC TC-3′. C_t_ values were given in relation to the endogenous housekeeping gene *Gapdh*, the expression of which was not affected by BMP4 treatment (see Figure S2B,C). FC calculations between treated and control samples were performed using the 2^−ΔΔCt^ method [35]. The two-tailed paired *t*-test was used for statistical analysis in BioRender Graph using R 4.2.2.

### 4.15. Immunofluorescence Staining

Immunofluorescence labeling was performed on BMP6-treated, BMPR1A-treated, or control cultured cells at day 4 of the long-term experiment using anti-pSMAD1/5 (Cell Signaling, 9516, RRID:AB_491015) and anti-pan-MYH (DSHB, MF 20, RRID:AB_2147781) as primary antibodies, followed by fluorescently labeled secondary antibodies (goat anti-mouse IgG (H+L) Alexa Fluor 594, Invitrogen, Darmstadt, Germany, A-11005, RRID:AB_141372, and F(ab’)2-goat anti-rabbit IgG (H+L) Alexa Fluor 488, Invitrogen, A-11070, RRID:AB_2534114). Briefly, the cells were fixed with 4% paraformaldehyde, quenched with 0.1 M glycine, permeabilized with cold 100% methanol, incubated with the primary antibodies overnight, incubated with the secondary antibodies for one hour, and counterstained with DAPI. Fluorescence was examined under the automated inverted microscope Axio Observer (Zeiss, Oberkochen, Germany) and images were acquired using the Zen software v3.4.

### 4.16. Automated Nuclei Counting and Calculation of the Fusion Index

Nuclei around fibers on day 4 of the long-term experiment were quantified using the Fiji/ImageJ 1.54 software (RRID:SCR_003070) [82]. For background subtraction, a background image was calculated from each frame with Fiji (see the macro on GitHub). To derive the corrected fluorescence intensity, the background image was subtracted from the respective frame. Regions of interest (here, the nuclei) were identified using the software’s “Nucleus Counter” plug-in. To calculate the fusion index, the number of nuclei inside fibers was divided by the total number of nuclei and multiplied by 100.

### 4.17. Code Availability

The scripts used to analyze all datasets are available on GitHub (RRID:SCR_002630) at https://github.com/BirtheLange/Lange_et_al_2026_BMP_Notch (accessed on 24 July 2026) and on Zenodo (RRID:SCR_004129) at https://doi.org/10.5281/zenodo.21389765 (accessed on 24 July 2026) for BigWig files used in the custom UCSC browser session.

## Data Availability

The original data presented in the study are openly available in the Gene Expression Omnibus (GEO) at GSE306430 (SuperSeries), composed of the SubSeries GSE159594 (RNA-seq 1 data), GSE127798 (microarray data), GSE305746 (RNA-seq 2 data), GSE339586 (inhibitor data, RNA-seq 3), and GSE305745 (CUT&Tag data), as well as https://doi.org/10.5281/zenodo.21389765 for BigWig files.

## Supplementary Materials

The following supporting information can be downloaded at: https://www.mdpi.com/article/10.3390/cells15161490/s1, Figure S1: Summary of RNA-based experiments; Figure S2: Validation of BMP-regulated genes using microarrays and pathway-focused PCR arrays; Figure S3: Short-term effects of BMP6 on gene expression of known target genes of canonical Notch signaling; Figure S4: Detailed example of the induction of HES1 protein by BMP6; Figure S5: Additional example of the induction of HES1 protein by BMP6; Figure S6: Comparison of RNA-seq experiments; Figure S7: Examples of pSMAD1/5/9 and SMAD4 CUT&Tag profiles; Table S1: List of the BMP signaling pathway (GO:0030509), Notch signaling pathway (GO:0007219), and skeletal muscle tissue development (GO:0007519) genes; Table S2: Changes in gene expression observed in the RNA-seq 1 experiment; Table S3: Changes in gene expression determined by microarray; Table S4: Changes in gene expression observed in the RNA-seq 2 experiment; Table S5: Differential peaks comparing SMAD binding by CUT&Tag in the presence/absence of BMP6.

## Abbreviations

The following abbreviations are used in this manuscript:

BH	Benjamini–Hochberg
BMP	Bone morphogenetic protein
BMPR1A	BMP receptor type 1A
CUT&Tag	Cleavage Under Targets and Tagmentation
DEG	Differentially expressed gene
DLL	Delta-like
FACS	Fluorescence-activated cell sorting
FBS	Fetal bovine serum
FC	Fold change
FDR	False discovery rate
FGF	Fibroblast growth factor
FPKM	Fragments per kilobase of transcript per million mapped reads
HGF	Hepatocyte growth factor
Id	Inhibitor of DNA binding
IDR	Irreproducible discovery rate
IGF-1	Insulin-like growth factor 1
JAG	Jagged
MACS^®^	Magnetic cell separation
MuSC	Muscle stem cell
Mymk	Myomaker
Myog	Myogenin
NICD	Notch intracellular domain
padj	Adjusted *p*-value
RIN	RNA integrity number
RNA-seq	RNA-sequencing
TGF-β	Transforming growth factor-β
TPM	Transcripts per million
TSS	Transcription start site
