# Supplementary material for: Stimulation of Adult Muscle Stem Cells with BMPs Results in Direct Activation of Notch Pathway Genes
Source: Cells. 2026 Aug 19;15(16):1490. doi: 10.3390/cells15161490 (PMC13511064; doi:10.3390/cells15161490)
Supplement: Supplementary file 1 [file cells-15-01490-s001.zip › 20260808_Lange_MuSC_Supplement.pdf]

Stimulation of Adult Muscle Stem Cells with BMPs Results in Direct Activation of Notch Pathway Genes

Birthe Katrin Alexandra Lange, Ioanna Polydorou, Viktoriia Huryn, Angelina M. Georgieva, Shanshan You, Thomas Müller, Susanne Morales-Gonzalez, Bettina Brandt, Carmen Birchmeier, Helge Amthor, Markus Schuelke

SUPPLEMENTARY INFORMATION

1. Supplementary Figures

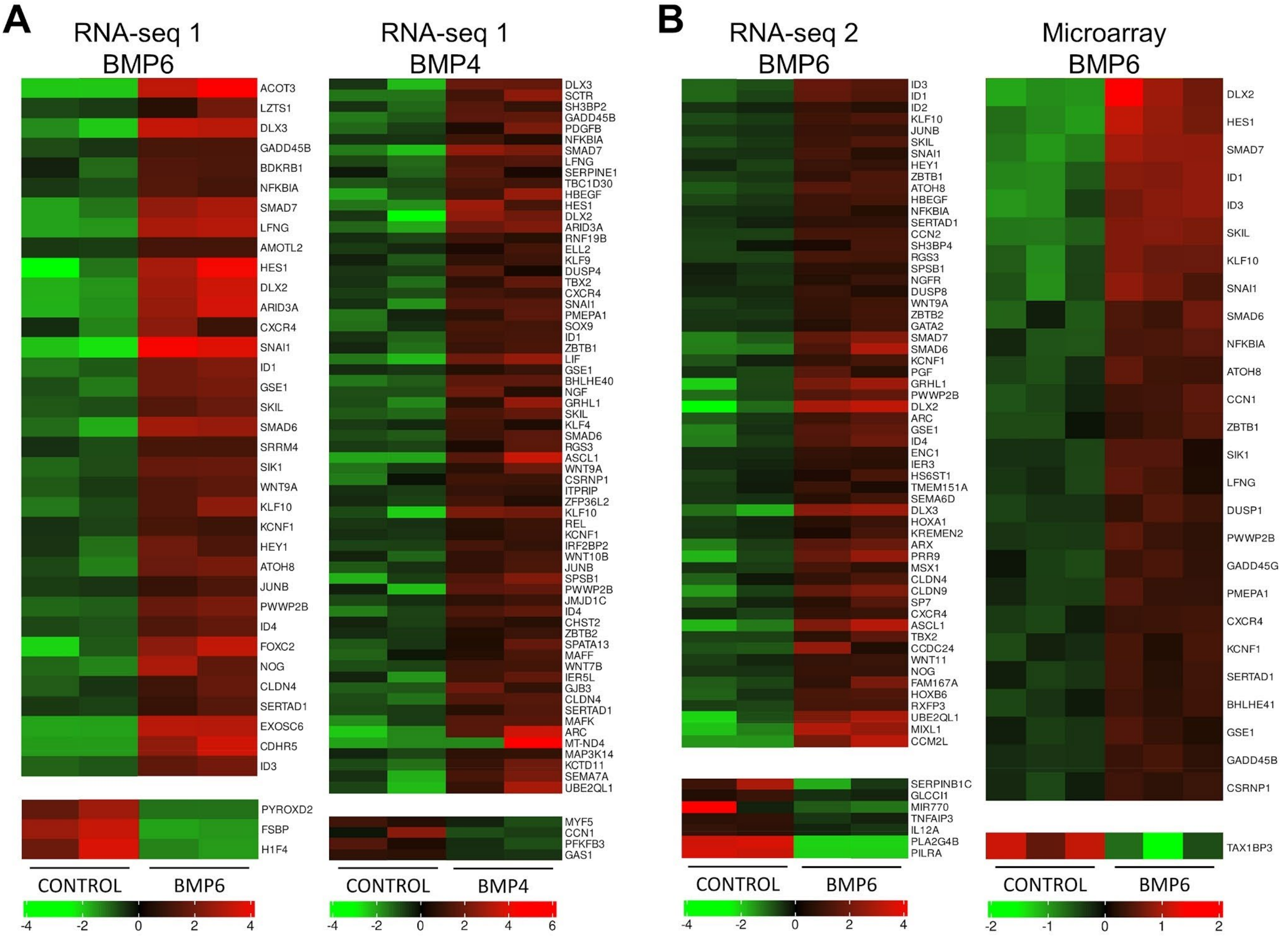

Figure S1. Summary of RNA-based experiments. Heatmaps of regulated genes identified in RNA-seq 1 after BMP6 and BMP4 stimulation (**A**), RNA-seq 2, and by microarray analysis (**B**). Color codes indicating the changes in expression are shown below.

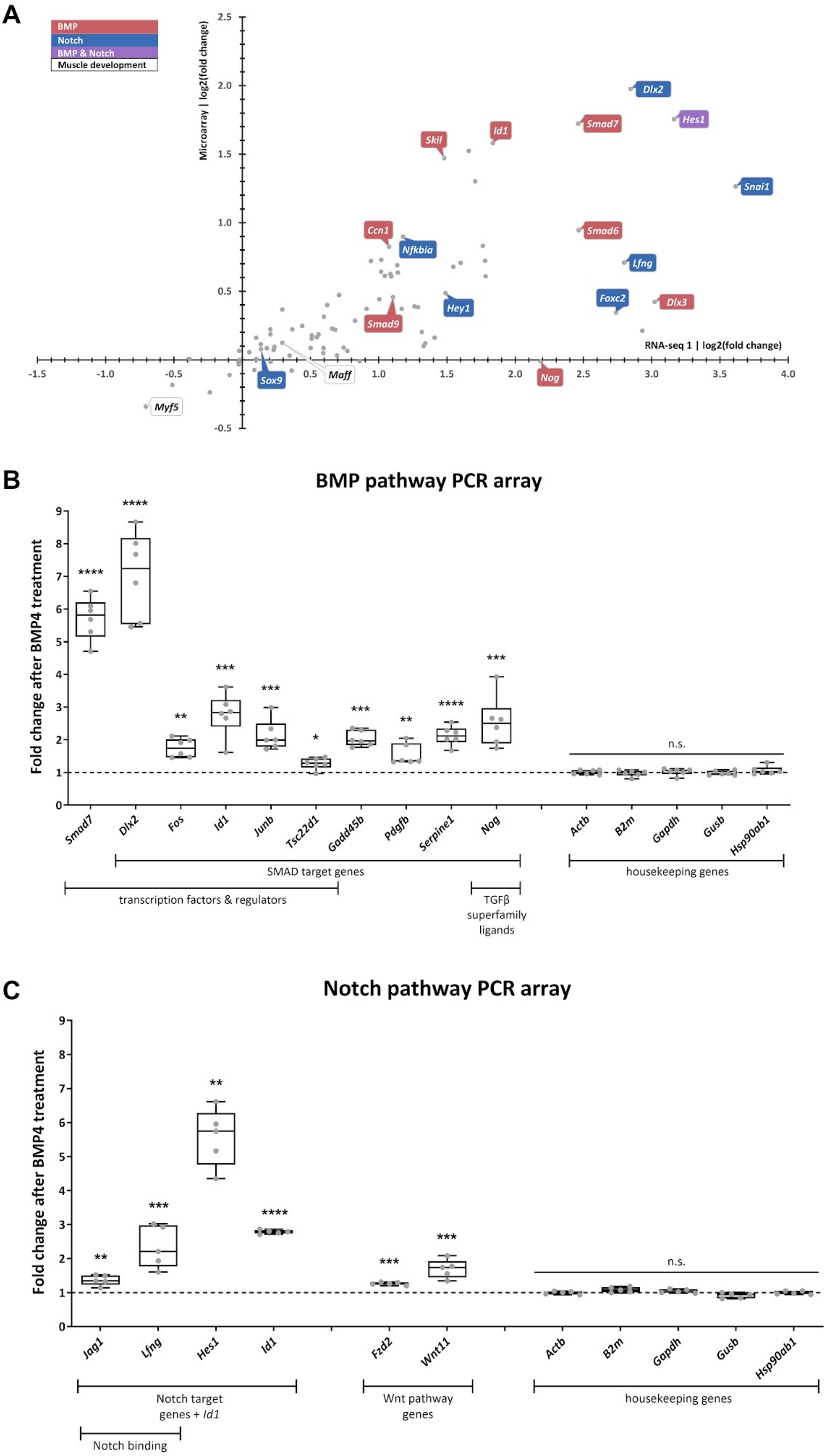
A
BMP
Notch
BMP & Notch
Muscle development
Microarray | log2(fold change)
RNA-seq 1 | log2(fold change)
Dlx2
Hes1
Smad7
Id1
Skil
Snai1
Ccn1
Nfkbia
Smad6
Lfng
Foxc2
Dlx3
Hey1
Smad9
Sox9
Maff
Nog
Myf5
B
BMP pathway PCR array
Fold change after BMP4 treatment
n.s.
Smad7
Dlx2
Fos
Id1
Junb
Tsc22d1
Gadd45b
Pdgfb
Serpine1
Nog
Actb
B2m
Gapdh
Gusb
Hsp90ab1
SMAD target genes
housekeeping genes
transcription factors & regulators
TGFβ superfamily ligands
C
Notch pathway PCR array
Fold change after BMP4 treatment
n.s.
Jag1
Lfng
Hes1
Id1
Fzd2
Wnt11
Actb
B2m
Gapdh
Gusb
Hsp90ab1
Notch target genes + Id1
Wnt pathway genes
housekeeping genes
Notch binding

Figure S2. Validation of BMP-regulated genes using microarrays and pathway-focused PCR arrays. (**A**) Regulation of BMP and Notch pathway genes in BMP6-stimulated, cultured mouse MuSCs analyzed by RNA-seq (X-axis) versus microarray (Y-axis). All genes that were regulated by BMP6 and/or BMP4 in RNA-seq 1 (FC ≥2.0 or ≤0.5, FDR(BH) <0.1) are represented by a grey dot. Genes of the BMP pathway and the Notch pathway are highlighted in color. Genes controlling skeletal muscle development are shown on a white background. N = 2 biological replicates per condition in the RNA-seq and n = 3 in the microarray experiments. (**B**, **C**) Pathway-focused quantitative PCR analysis of BMP4 regulated BMP (**B**) and Notch (**C**) pathway genes. DEGs are grouped according to their functions. The housekeeping genes *Actb*, *B2m*, *Gapdh*, *Gusb*, and *Hsp90ab1* were used for normalization. FC calculations were performed using the $2^{-\Delta\Delta Ct}$ method [35]. Median, interquartile, and interdecile values are indicated in the boxplots. The paired two-tailed t-test was used for statistical analysis of the resulting FCs. Significance levels, FDR(BH): *, adjusted p-value (padj) <0.1; **, padj <0.05; ***, padj <0.01; ****, padj <0.001. N = 6 biological replicates per condition for the BMP pathway array and n = 5 for the Notch pathway array.

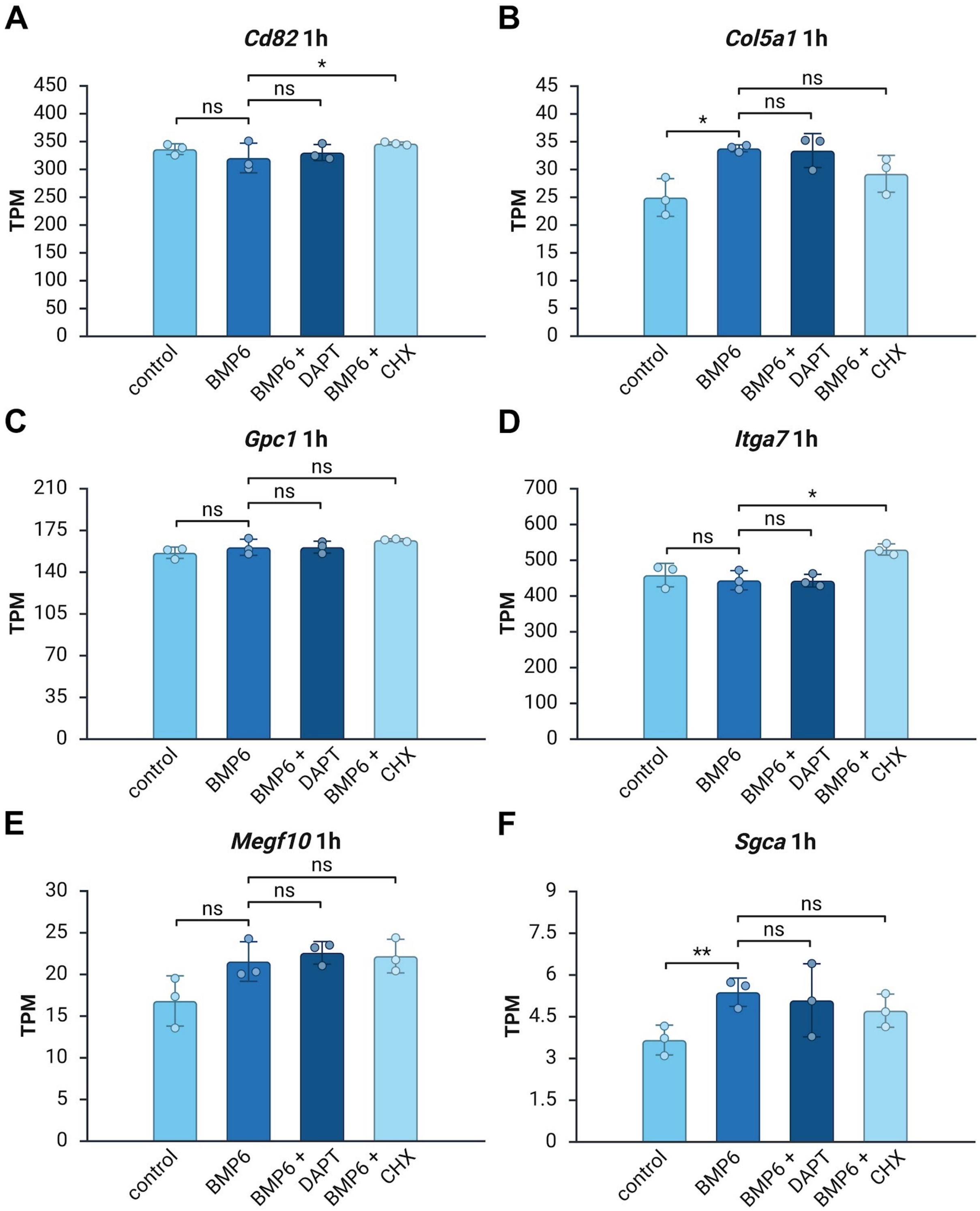

Figure S3. Short-term effects of BMP6 on gene expression of known target genes of canonical Notch signaling. TPMs of known direct Notch target genes with or w/o stimulation of MuSCs by BMP6 were determined by RNA-seq. Cells were starved for six hours in the presence of the antagonistic BMP receptor fragment BMPR1A-Fc, and subsequently exposed for one and three hours to BMP6, BMP6 plus DAPT (Notch signaling inhibitor), or BMP6 plus cycloheximide (protein synthesis inhibitor). The panels depict analyses of Notch target genes *Cd82*, *Col5a1*, *Gpc1*, *Itga7*, *Megf10*, and *Sgca* (**A–F**). The two-tailed paired t-test was used for statistical analyses. Significance levels: *, $p < 0.05$; **, $p < 0.01$; ns, not significant. Values are shown as mean ± SD of n = 3 biological replicates per condition.

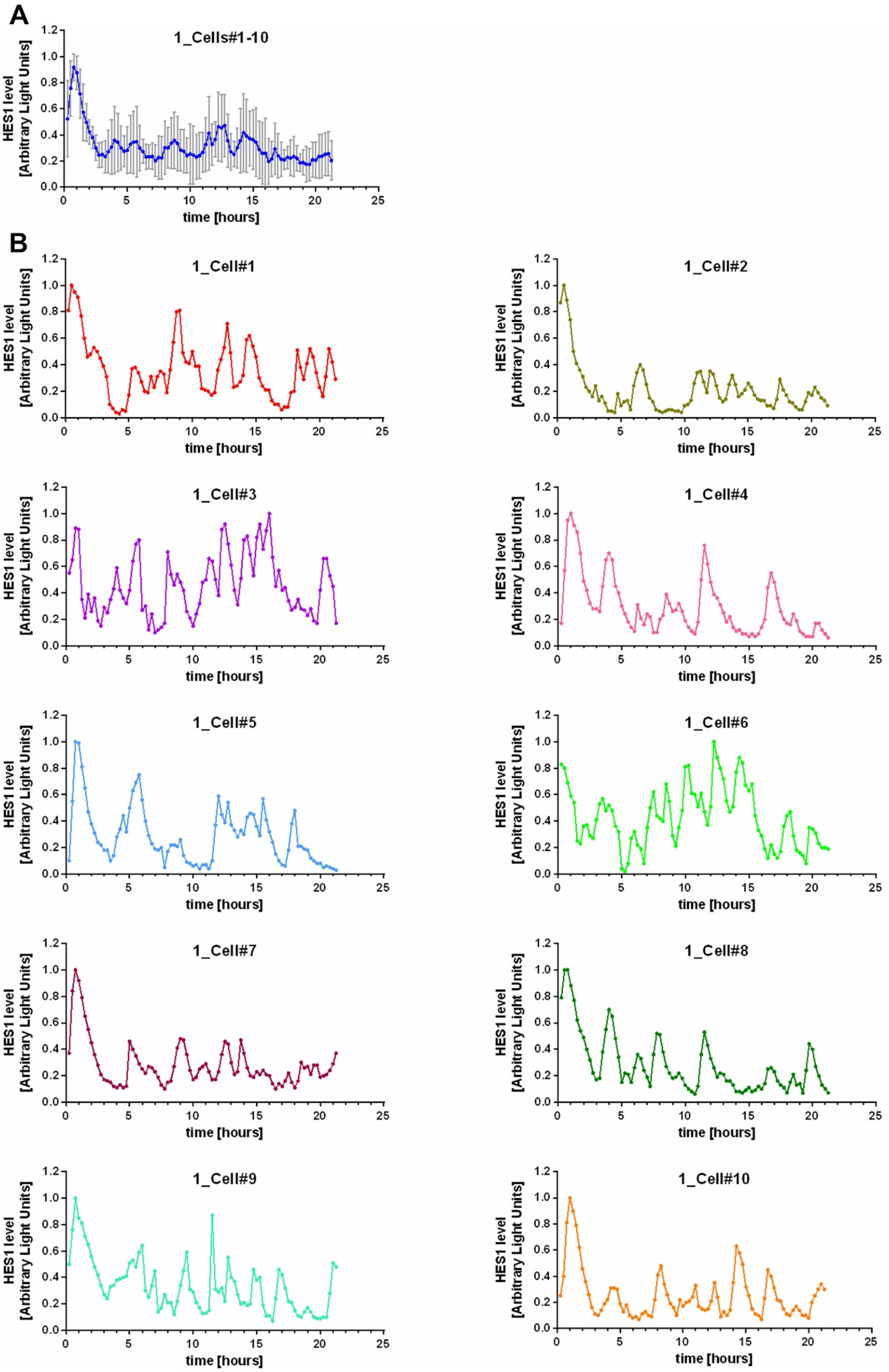

Figure S4. Detailed example of the induction of HES1 protein by BMP6. Luciferase activity produced in response to BMP6 in MuSCs of the *Hes1-nLuc* strain (luciferase coding sequences fused to the endogenous *Hes1* locus; HES1-nLuc fusion protein is produced by the gene). Life imaging of cells was used to record the luciferase signals over prolonged periods. (**A**) Average ± SD of luciferase signals obtained from a total of 10 individual cells. (**B**) Luciferase activity in response to BMP6 in 10 individual cells. The same replicate as in **Figure 4** is shown with all 10 individual cells; n = 5 biological replicates. See also **Figure S5** for an additional replicate.

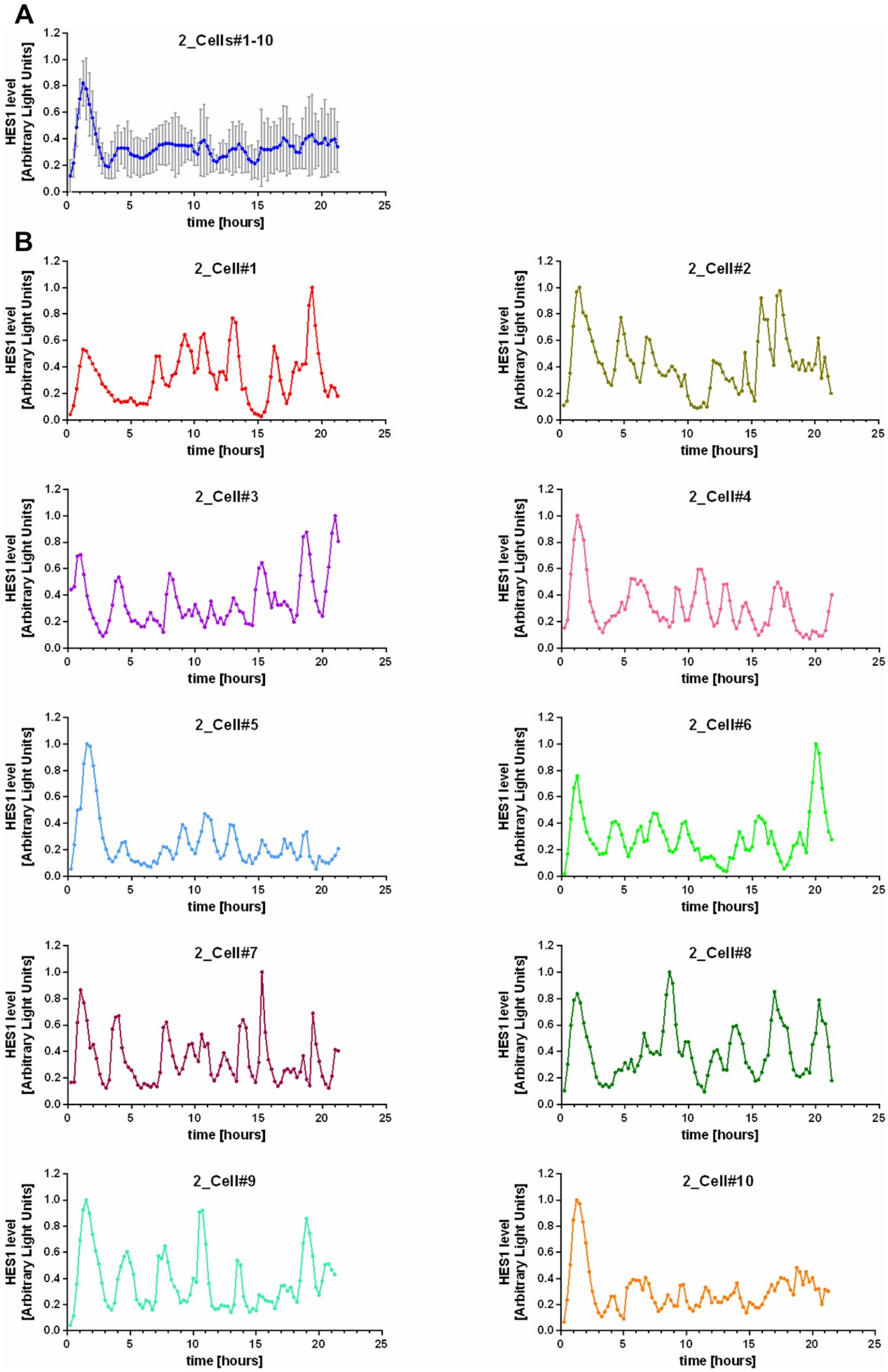

Figure S5. Additional example of the induction of HES1 protein by BMP6. Luciferase activity produced in response to BMP6 in MuSCs of the *Hes1-nLuc* strain (luciferase coding sequences fused to the endogenous *Hes1* locus; HES1-nLuc fusion protein is produced by the gene). Life imaging of cells was used to record the luciferase signals over prolonged periods. (**A**) Average ± SD of luciferase signals obtained from a total of 10 individual cells. (**B**) Luciferase activity in response to BMP6 in 10 individual cells. A second out of n = 5 biological replicates is shown. See also **Figure S4** for an additional replicate.

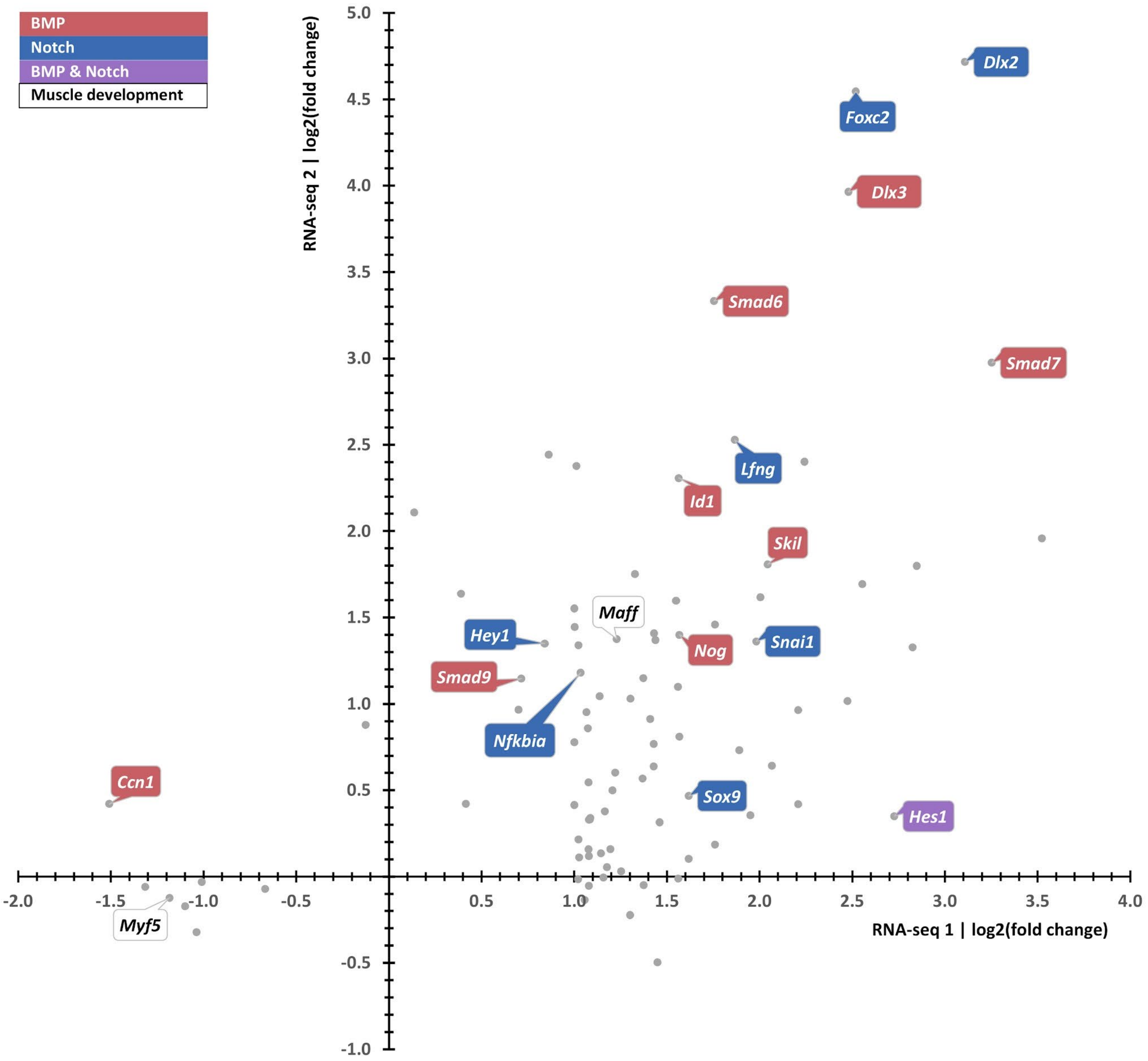

Figure S6. Comparison of RNA-seq experiments. Regulation of BMP and Notch pathway genes in BMP6-stimulated, cultured mouse MuSCs analyzed by RNA-seq 1 (X-axis) versus RNA-seq 2 (Y-axis). All genes that were regulated by BMP6 and/or BMP4 in RNA-seq 1 (FC ≥2.0 or ≤0.5, FDR(BH) <0.1) are represented by a grey dot. Genes of the BMP pathway and the Notch pathway are highlighted in color. Genes controlling skeletal muscle development are shown on a white background. N = 2 biological replicates per condition.

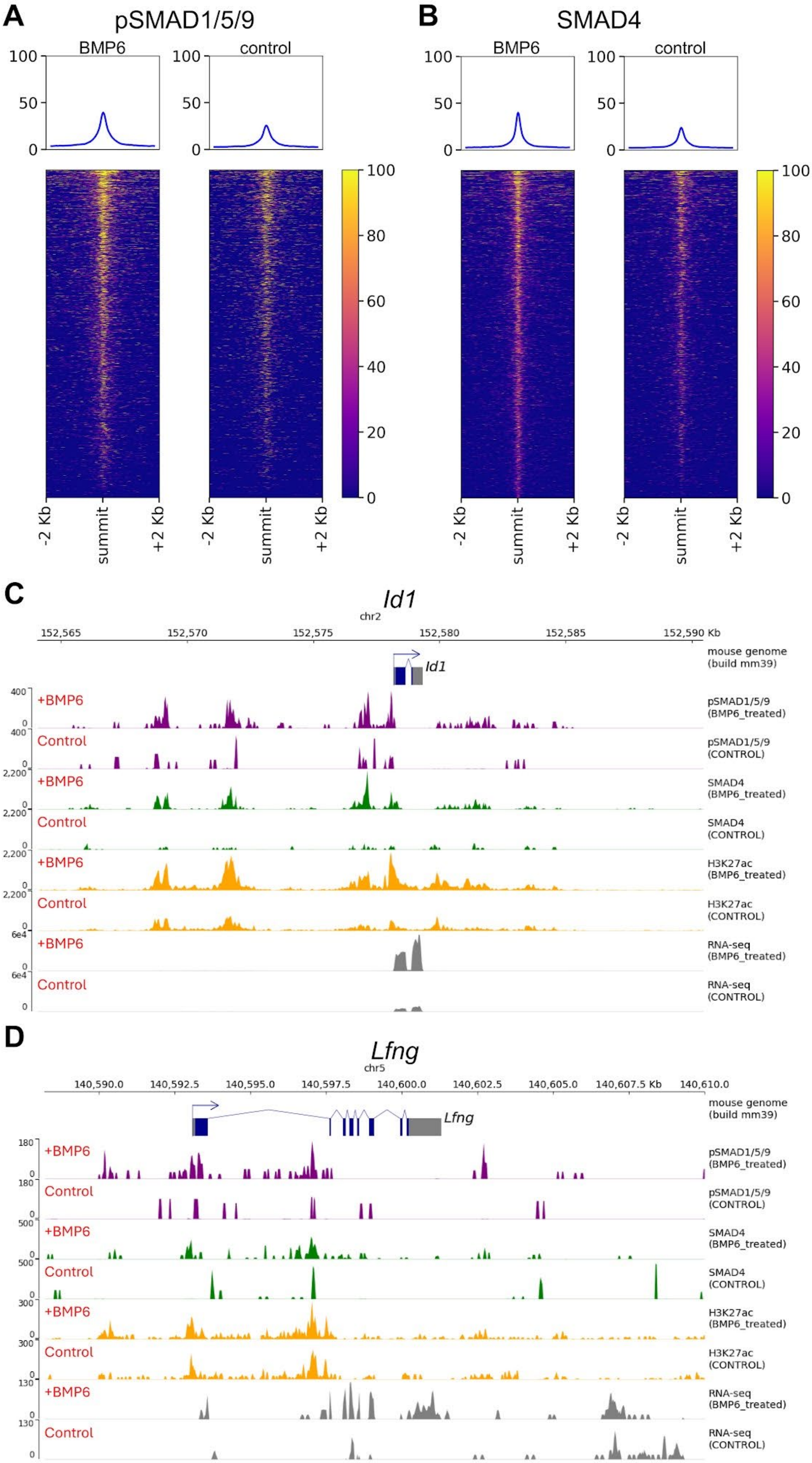
A
pSMAD1/5/9
BMP6
control
B
SMAD4
BMP6
control
100
50
0
80
60
40
20
-2 Kb
summit
+2 Kb
C
Id1
chr2
152,565
152,570
152,575
152,580
152,585
152,590 Kb
mouse genome (build mm39)
+BMP6
Control
pSMAD1/5/9 (BMP6_treated)
pSMAD1/5/9 (CONTROL)
SMAD4 (BMP6_treated)
SMAD4 (CONTROL)
H3K27ac (BMP6_treated)
H3K27ac (CONTROL)
RNA-seq (BMP6_treated)
RNA-seq (CONTROL)
400
2,200
6e4
D
Lfng
chr5
140,590.0
140,592.5
140,595.0
140,597.5
140,600.0
140,602.5
140,605.0
140,607.5 Kb
140,610.0
180
500
300
130

Figure S7. Examples of pSMAD1/5/9 and SMAD4 CUT&Tag profiles. (**A**, **B**) Average profiles and heatmaps of pSMAD1/5/9 (**A**) and SMAD4 (**B**) CUT&Tag peaks from BMP6-treated versus control cells centered around the peak summit. The second out of n = 2 biological replicates is shown. (**C**, **D**) Binding of pSMAD1/5/9 and SMAD4 to the promoters of *Id1* (**C**) and *Lfng* (**D**). Shown are the CUT&Tag traces of pSMAD1/5/9 (BMP6 versus control) in purple, SMAD4 traces (BMP6 versus control) in green, as well as analysis of H3K27ac (BMP6 versus control) in orange. RNA-seq traces (BMP6 versus control) are depicted in grey at the bottom. One out of n = 2 biological replicates is shown.

2. Supplementary Tables

Table S1. List of the BMP signaling pathway (GO:0030509), Notch signaling pathway (GO:0007219), and skeletal muscle tissue development (GO:0007519) genes.

Table S2. Changes in gene expression observed in the RNA-seq 1 experiment. (**A**) Raw counts per gene or unassigned fragment assembly. (**B**) DESeq2 output. (**C**) DESeq2 output filtered for genes and unassigned fragment assemblies that were significantly different in BMP-treated and control samples (FDR(BH) <0.1). (**D**) Genes and unassigned fragment assemblies that fulfill the FDR(BH) requirements and display an FC ≥2.0 or ≤0.5 after BMP6 and/or BMP4 stimulation. (**E**) Final list of DEGs filtering out gene names starting with "Gm" or "Mir" or ending in "Rik", "os", or "-ps" and unassigned fragment assemblies.

Table S3. Changes in gene expression determined by microarray. (**A**) Normalized intensities per probe. (**B**) limma output. (**C**) limma output filtered for genes that were significantly different in BMP-treated and control samples (FDR(BH) <0.1). (**D**) Genes that fulfill the FDR(BH) requirements and display an FC ≥1.41 or ≤0.71 after BMP6 stimulation.

Table S4. Changes in gene expression observed in the RNA-seq 2 experiment. (**A**) Raw counts per gene or unassigned fragment assembly. (**B**) DESeq2 output. (**C**) DESeq2 output filtered for genes and unassigned fragment assemblies that were significantly different in BMP-treated and control samples (FDR(BH) <0.1). (**D**) Genes and unassigned fragment assemblies that fulfill the FDR(BH) requirements and display an FC ≥2.0 or ≤0.5 after BMP6 stimulation. (**E**) Final list of DEGs filtering out gene names starting with "Gm" or "Mir" or ending in "Rik", "os", or "-ps" and unassigned fragment assemblies.

Table S5. Differential peaks comparing SMAD binding by CUT&Tag in the presence/absence of BMP6. (**A**) All differential pSMAD1/5/9 peaks. (**B**) All differential SMAD4 peaks. (**C**) All differential pSMAD1/5/9 peaks within -1.5 kb and +0.5 kb of the TSS of the reference transcript of the DEGs from RNA-seq 1. (**D**) All differential SMAD4 peaks within -1.5 kb and +0.5 kb of the TSS of the reference transcript of the DEGs from RNA-seq 1. (**E**) All differential pSMAD1/5/9 peaks within 20 kb of the untranslated regions of the reference transcript of the DEGs from RNA-seq 1. (**F**) All differential SMAD4 peaks within 20 kb of the untranslated regions of the reference transcript of the DEGs from RNA-seq 1.
